# Author Correction: Muskelin is a substrate adaptor of the highly regulated *Drosophila* embryonic CTLH E3 ligase

**DOI:** 10.1038/s44319-026-00871-9

**Published:** 2026-07-17

**Authors:** Chloe A Briney, Jesslyn C Henriksen, Chenwei Lin, Lisa A Jones, Leif Benner, Addison B Rains, Roxana Gutierrez, Philip R Gafken, Olivia S Rissland

**Affiliations:** 1 Department of Biochemistry and Molecular Genetics, University of Colorado Anschutz Medical Campus, Aurora, CO 80045 USA; 2 RNA Bioscience Initiative, University of Colorado Anschutz Medical Campus, Aurora, CO 80045 USA; 3 Proteomics & Metabolomics Shared Resource, Fred Hutchinson Cancer Center, Seattle, WA 98109 USA; 4 Section of Developmental Genomics, Laboratory of Biochemistry and Genetics, National Institute of Diabetes and Digestive and Kidney Diseases, National Institutes of Health, Bethesda, MD 20892 USA

## Abstract

**Figure:**
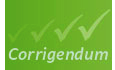

**Correction to:** *EMBO Reports* (2025) 26:1647–1669. 10.1038/s44319-025-00397-6 | Published online 20 February 2025

The authors contacted the journal after identifying an error in the Methods section of the published article. The journal has reviewed the changes, and the following modification has been made:

**Methods section corrected**.

Author statement:

In the Methods section, under the heading “Denaturing cross-linking immunoprecipitations” is corrected from:

Lysates were incubated with 50 mM DTSP (Millipore Sigma) or DMSO (Millipore Sigma) for 3 h at 4 °C rotating. The reaction was quenched by adding Tris-HCl pH 7.5 to 20 mM. An equal volume 2× denaturing lysis buffer (2× PBS, 2% Triton X-100, 2% sodium deoxycholate, 2% SDS) was added followed by immunoprecipitation with anti-ME31B (Boster), anti-Muskelin (Boster), or IgG (Abcam) for 1 h at 4 °C. EZView protein G affinity beads (Sigma) were washed with lysis buffer A 3×. The lysate-antibody mixtures were added to 25 µL of washed protein G beads and rotated for 1 h at 4 °C. Beads were washed 3× with denaturing wash buffer (50 mM Tris pH 8, 1 M NaCl, 1% NP-40, 0.05% sodium deoxycholate, 0.1% SDS) and transferred to new tubes. Samples were reduced with DTT to reverse crosslink prior to western blotting or mass spectrometry analysis.

To: Changes in bold.

Lysates were incubated with **0.2** **mM** DTSP (Millipore Sigma) or DMSO (Millipore Sigma) for 3h at 4 °C rotating. The reaction was quenched by adding Tris-HCl pH 7.5 to 20 mM. An equal volume 2× denaturing lysis buffer (2× PBS, 2% Triton X-100, 2% sodium deoxycholate, 2% SDS) was added followed by immunoprecipitation with anti-ME31B (Boster), anti-Muskelin (Boster), or IgG (Abcam) for 1 h at 4 °C. EZView protein G affinity beads (Sigma) were washed with lysis buffer A 3×. The lysate-antibody mixtures were added to 25 µL of washed protein G beads and rotated for 1 h at 4 °C. Beads were washed 3× with denaturing wash buffer (50 mM Tris pH 8, 1 M NaCl, 1% NP-40, 0.05% sodium deoxycholate, 0.1% SDS) and transferred to new tubes. Samples were reduced with DTT to reverse crosslink prior to western blotting or mass spectrometry analysis.

This change reflects the correct DTSP concentration used in the experiment.

All authors agree to this correction and apologise for any inconvenience caused.

