# Supplementary figures and images for: Muskelin is a substrate adaptor of the highly regulated Drosophila embryonic CTLH E3 ligase
Source: EMBO Rep. 2025 Feb 20;26(6):1647–69. doi: 10.1038/s44319-025-00397-6 (PMC11933467; doi:10.1038/s44319-025-00397-6)

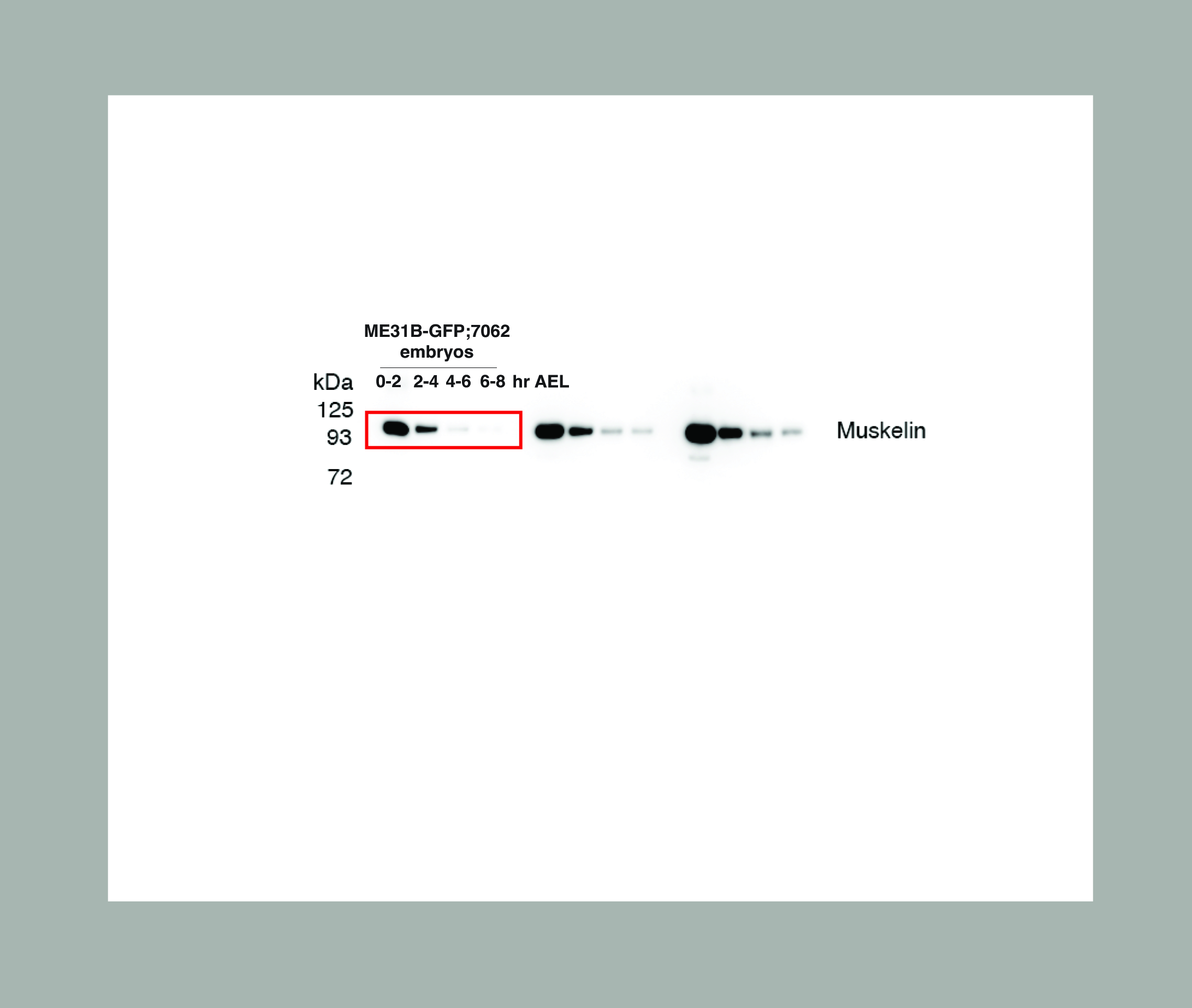

Supplement: Supplementary file 9 — Source data Fig. 1 [file 44319_2025_397_MOESM9_ESM.zip › Figure 1/1B/western muskelin.tif]

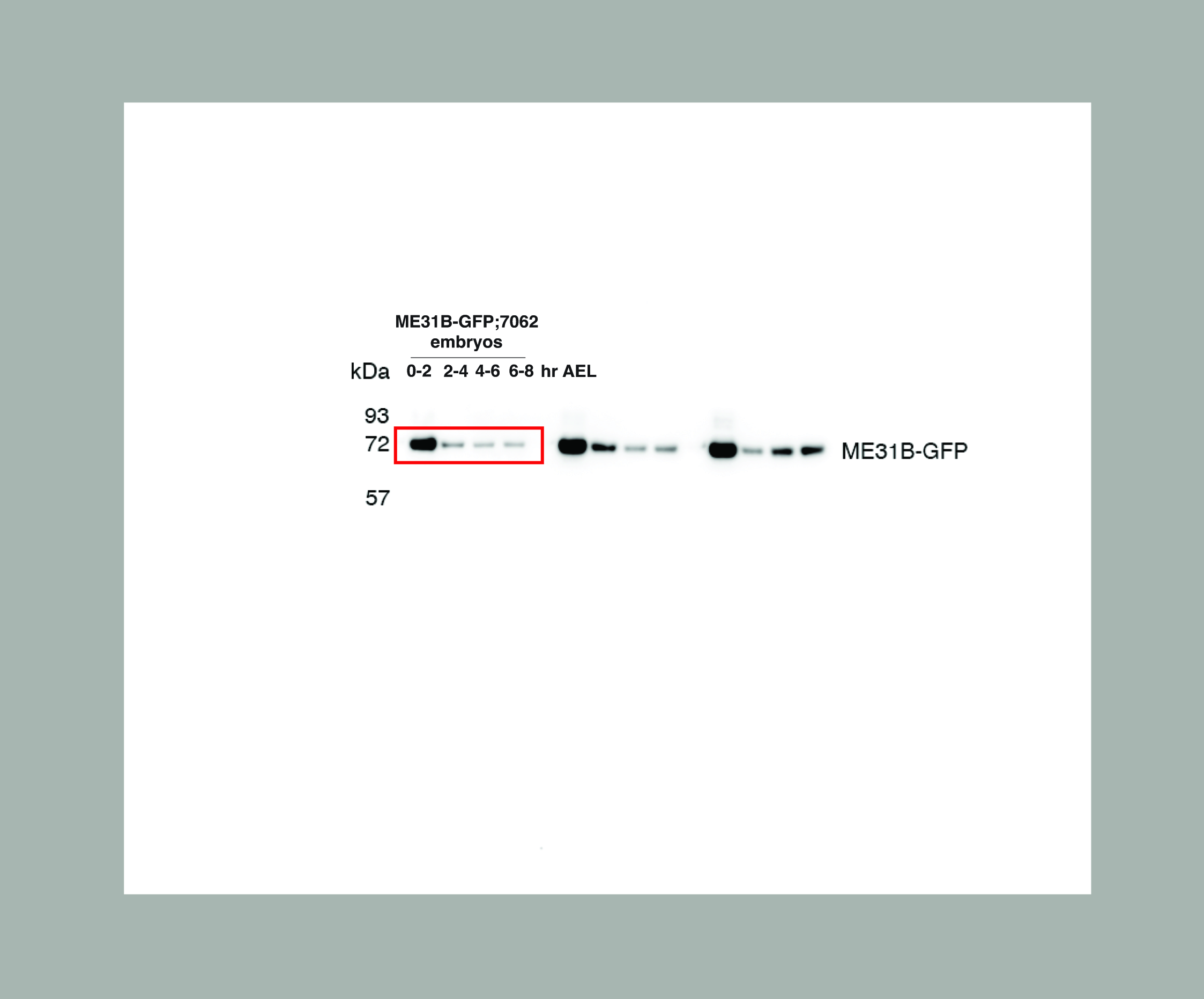

Supplement: Supplementary file 9 — Source data Fig. 1 [file 44319_2025_397_MOESM9_ESM.zip › Figure 1/1B/western gfp.tif]

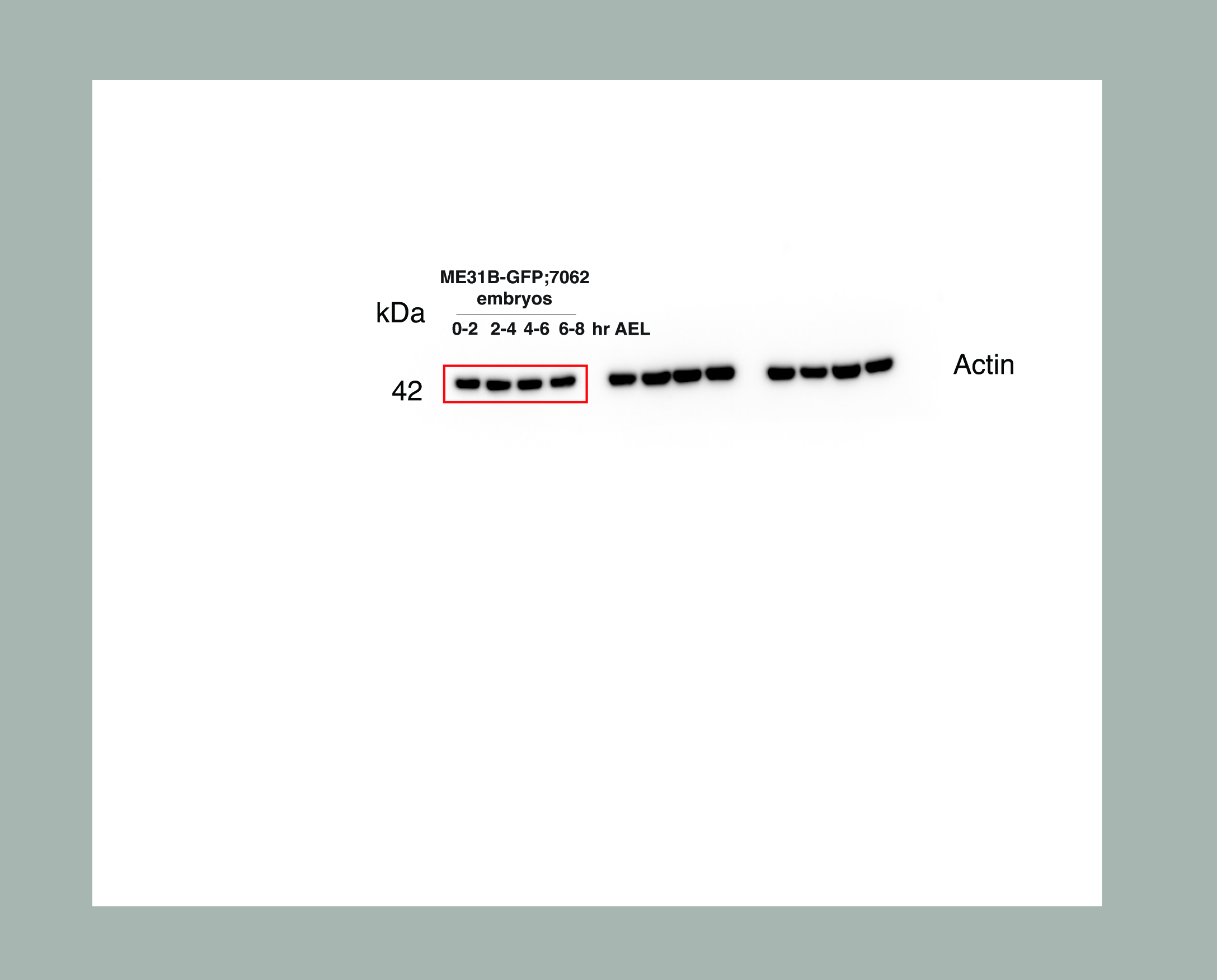

Supplement: Supplementary file 9 — Source data Fig. 1 [file 44319_2025_397_MOESM9_ESM.zip › Figure 1/1B/western actin.tif]

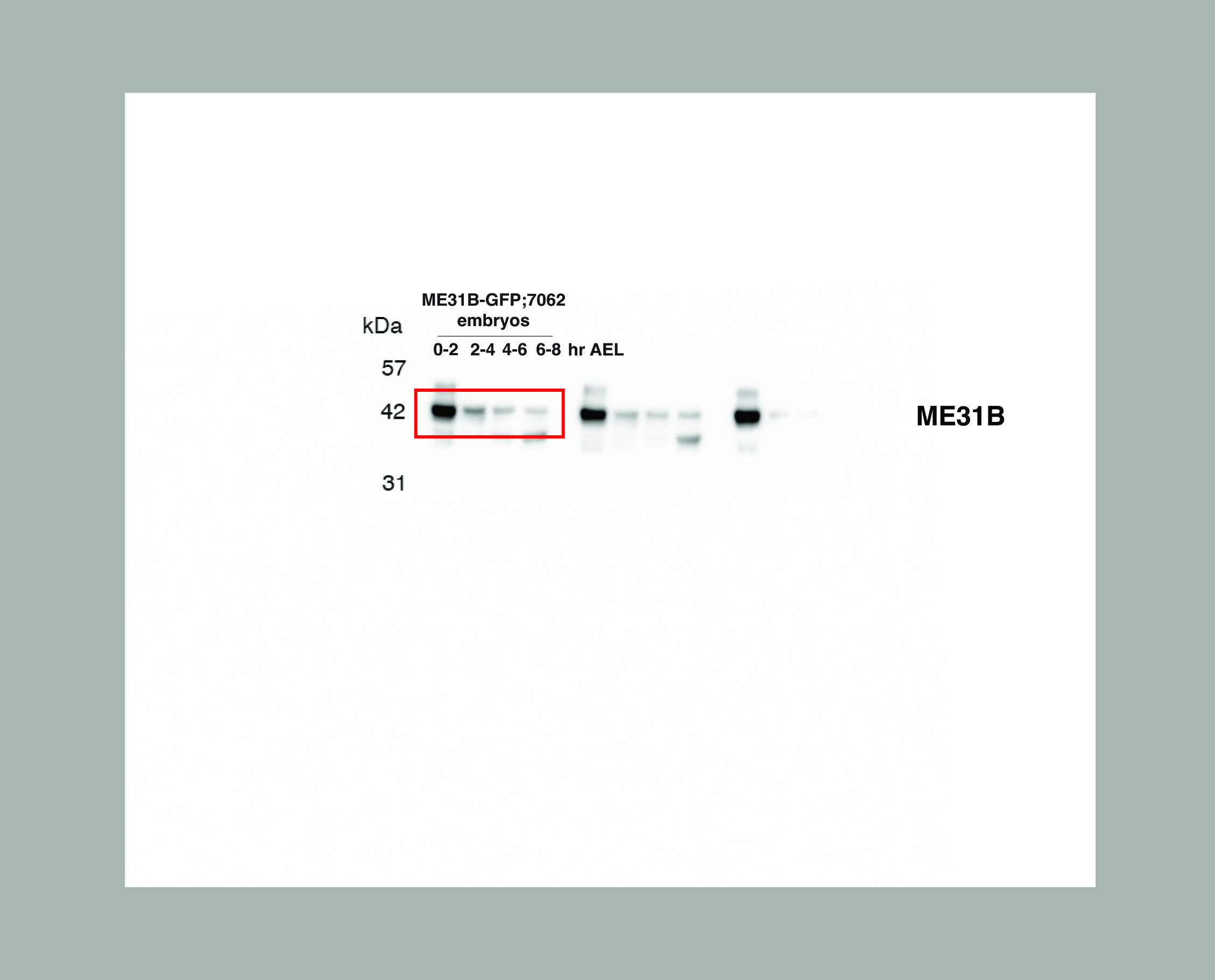

Supplement: Supplementary file 9 — Source data Fig. 1 [file 44319_2025_397_MOESM9_ESM.zip › Figure 1/1B/western me31b.tif]

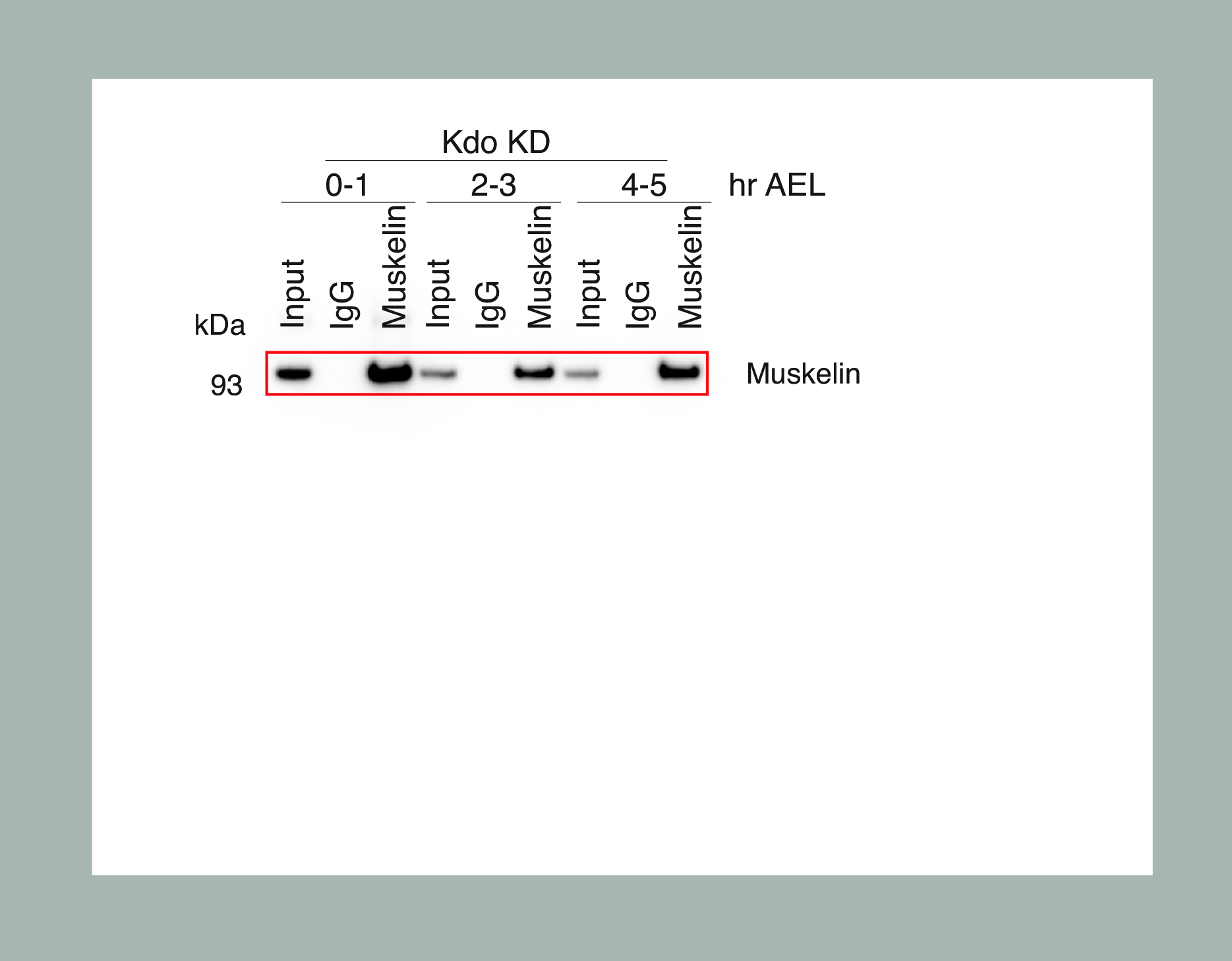

Supplement: Supplementary file 9 — Source data Fig. 1 [file 44319_2025_397_MOESM9_ESM.zip › Figure 1/1E/western muskelin kdokd.tif]

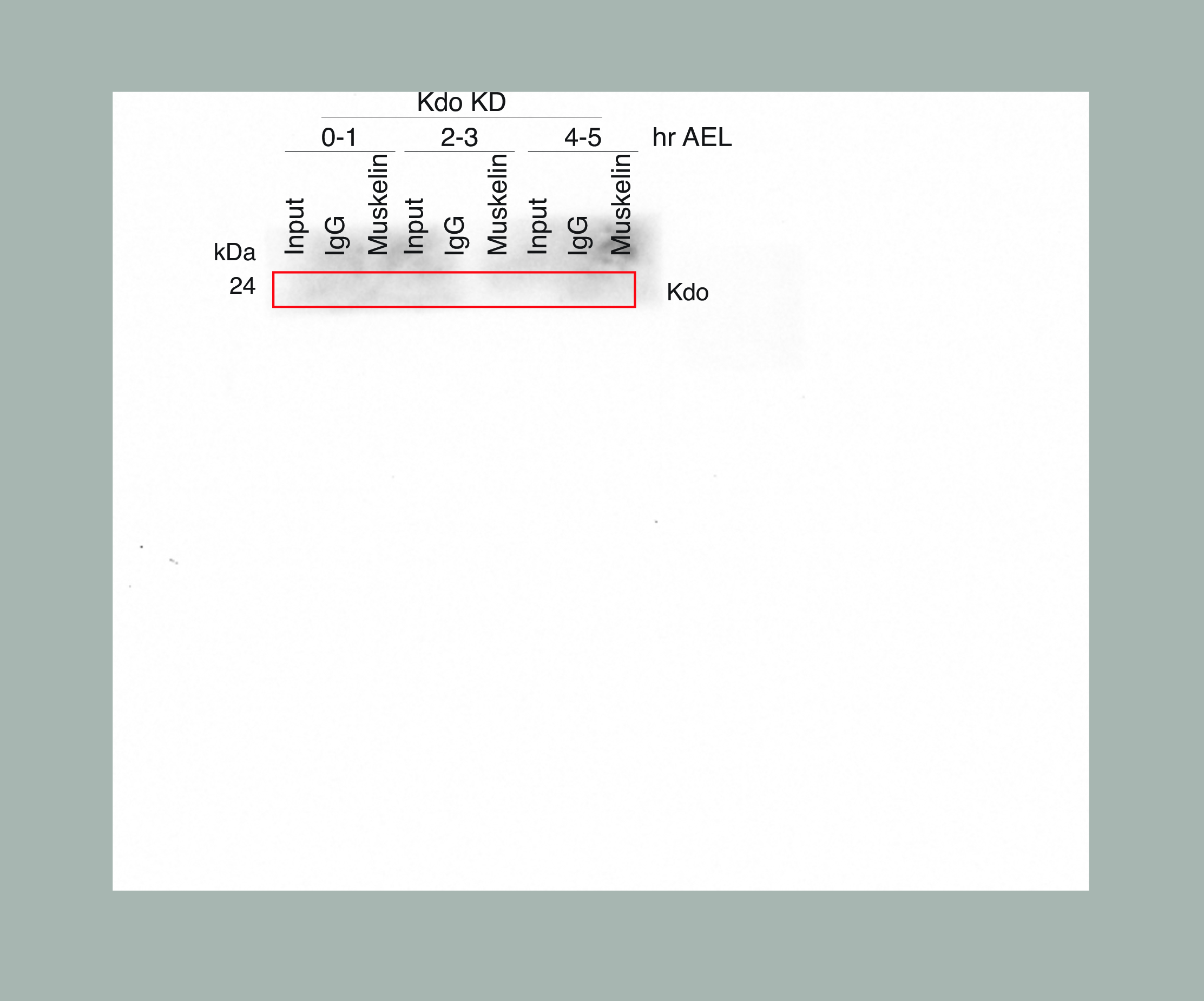

Supplement: Supplementary file 9 — Source data Fig. 1 [file 44319_2025_397_MOESM9_ESM.zip › Figure 1/1E/western kdo kdokd.tif]

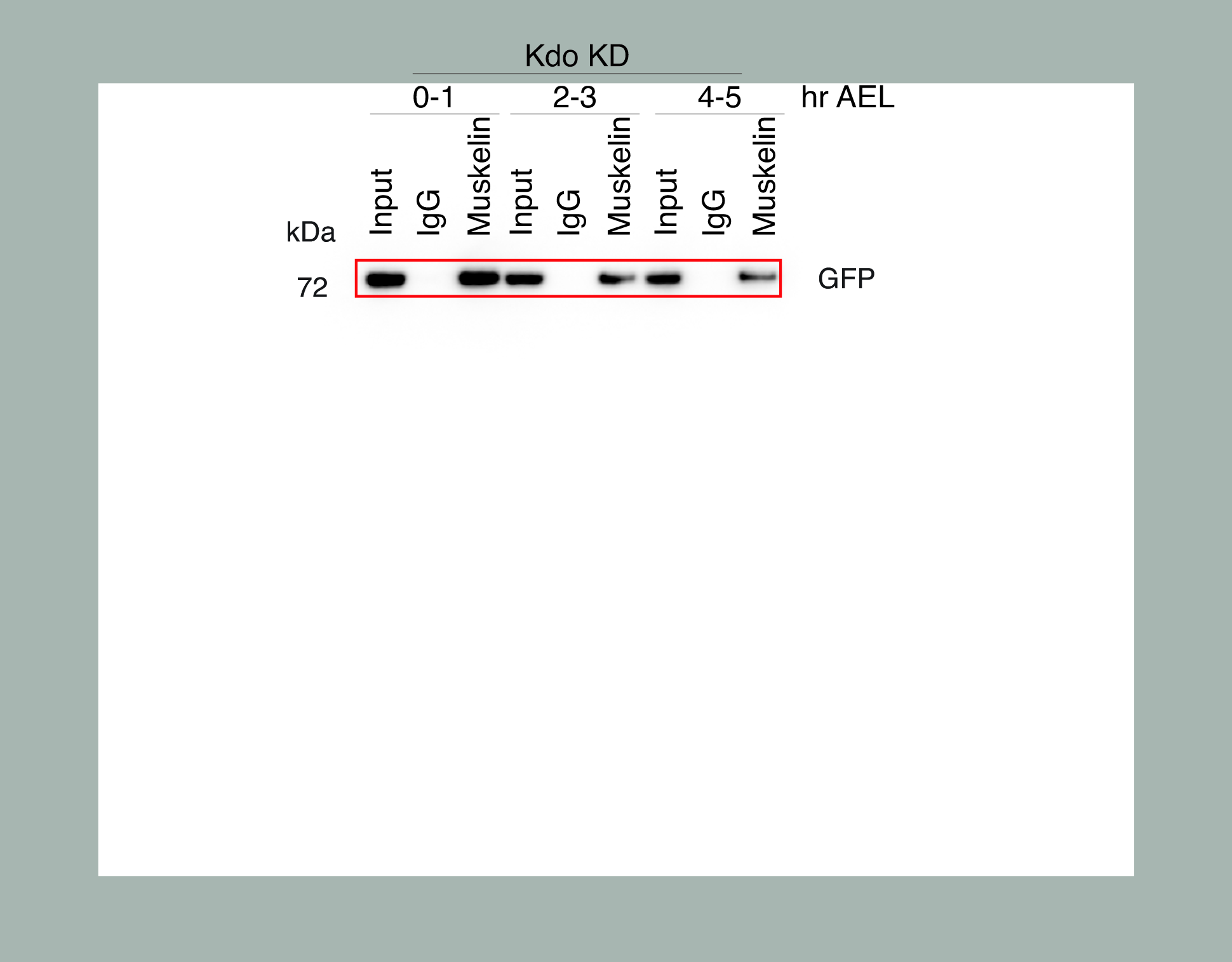

Supplement: Supplementary file 9 — Source data Fig. 1 [file 44319_2025_397_MOESM9_ESM.zip › Figure 1/1E/western gfp kdokd.tif]

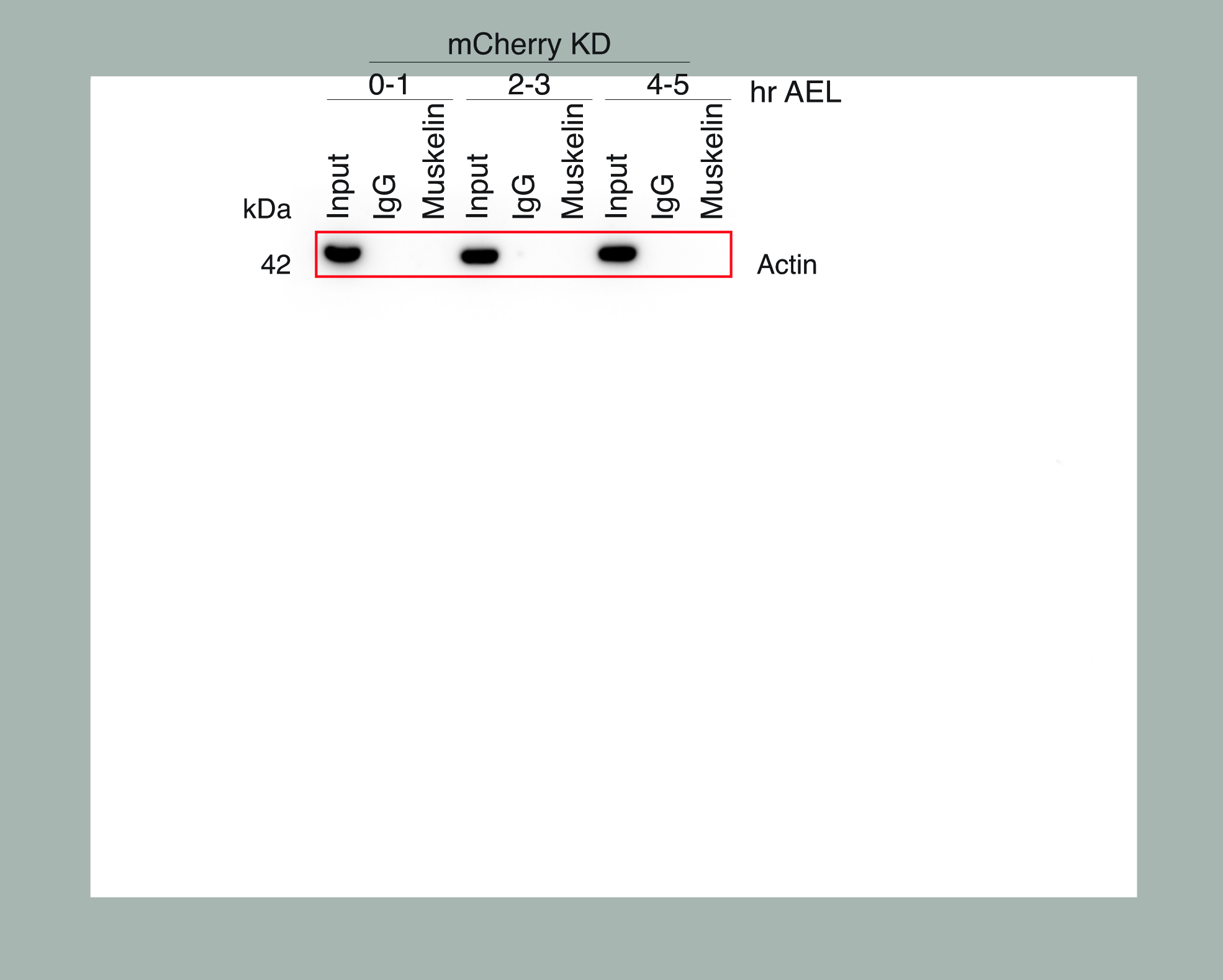

Supplement: Supplementary file 9 — Source data Fig. 1 [file 44319_2025_397_MOESM9_ESM.zip › Figure 1/1E/western actin mcherrykd.tif]

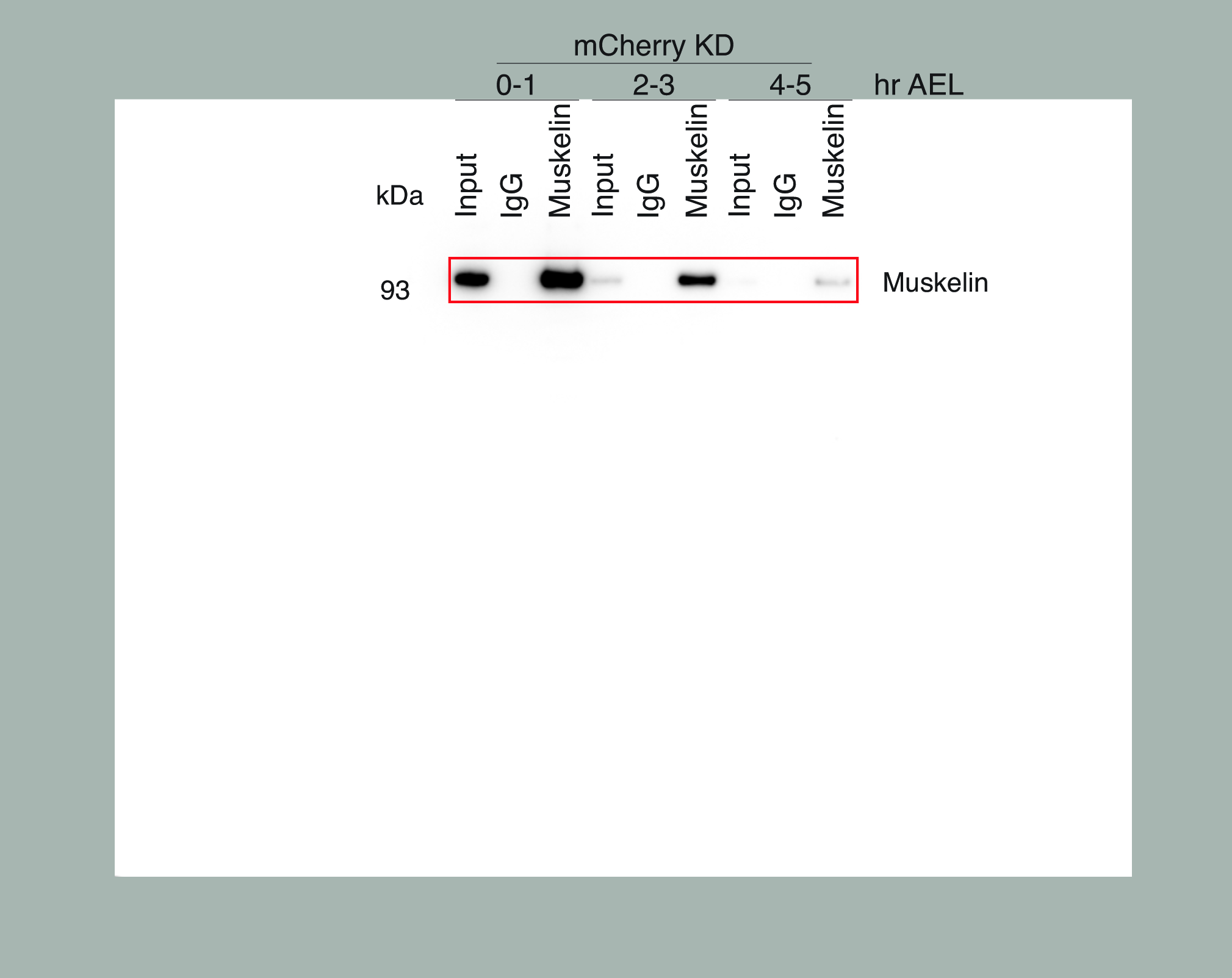

Supplement: Supplementary file 9 — Source data Fig. 1 [file 44319_2025_397_MOESM9_ESM.zip › Figure 1/1E/western muskelin mcherrykd.tif]

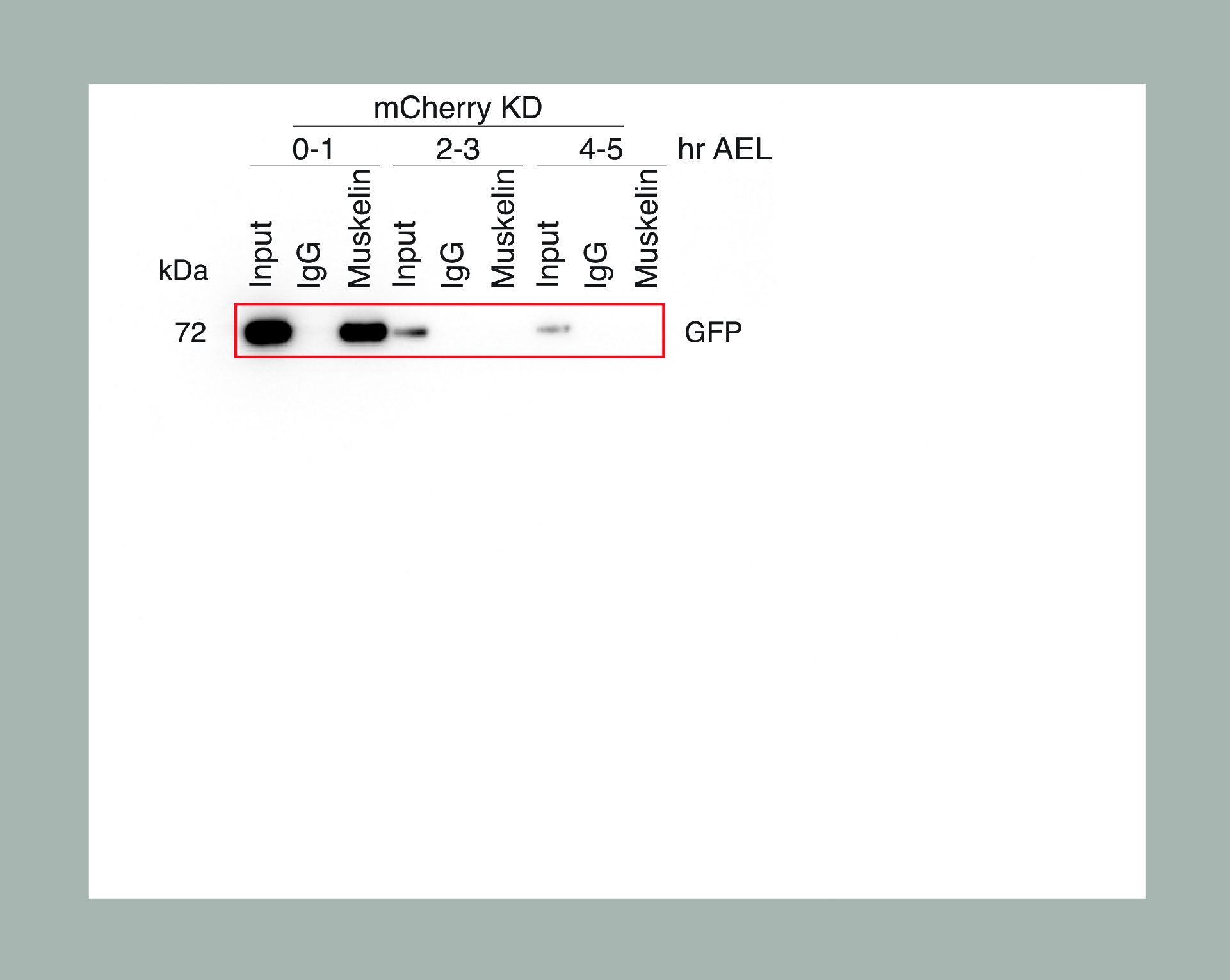

Supplement: Supplementary file 9 — Source data Fig. 1 [file 44319_2025_397_MOESM9_ESM.zip › Figure 1/1E/western gfp mcherrykd.tif]

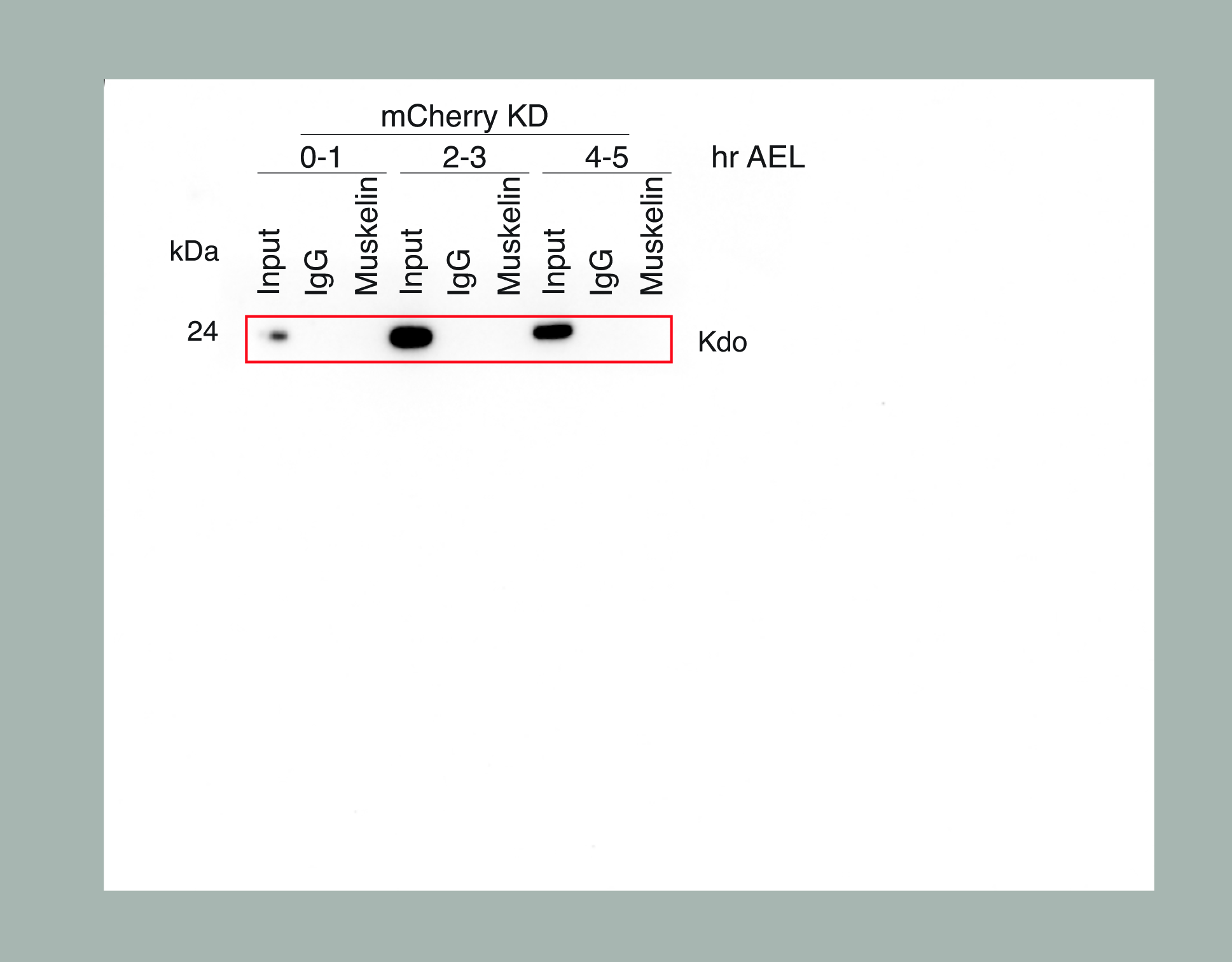

Supplement: Supplementary file 9 — Source data Fig. 1 [file 44319_2025_397_MOESM9_ESM.zip › Figure 1/1E/western kdo mcherrykd.tif]

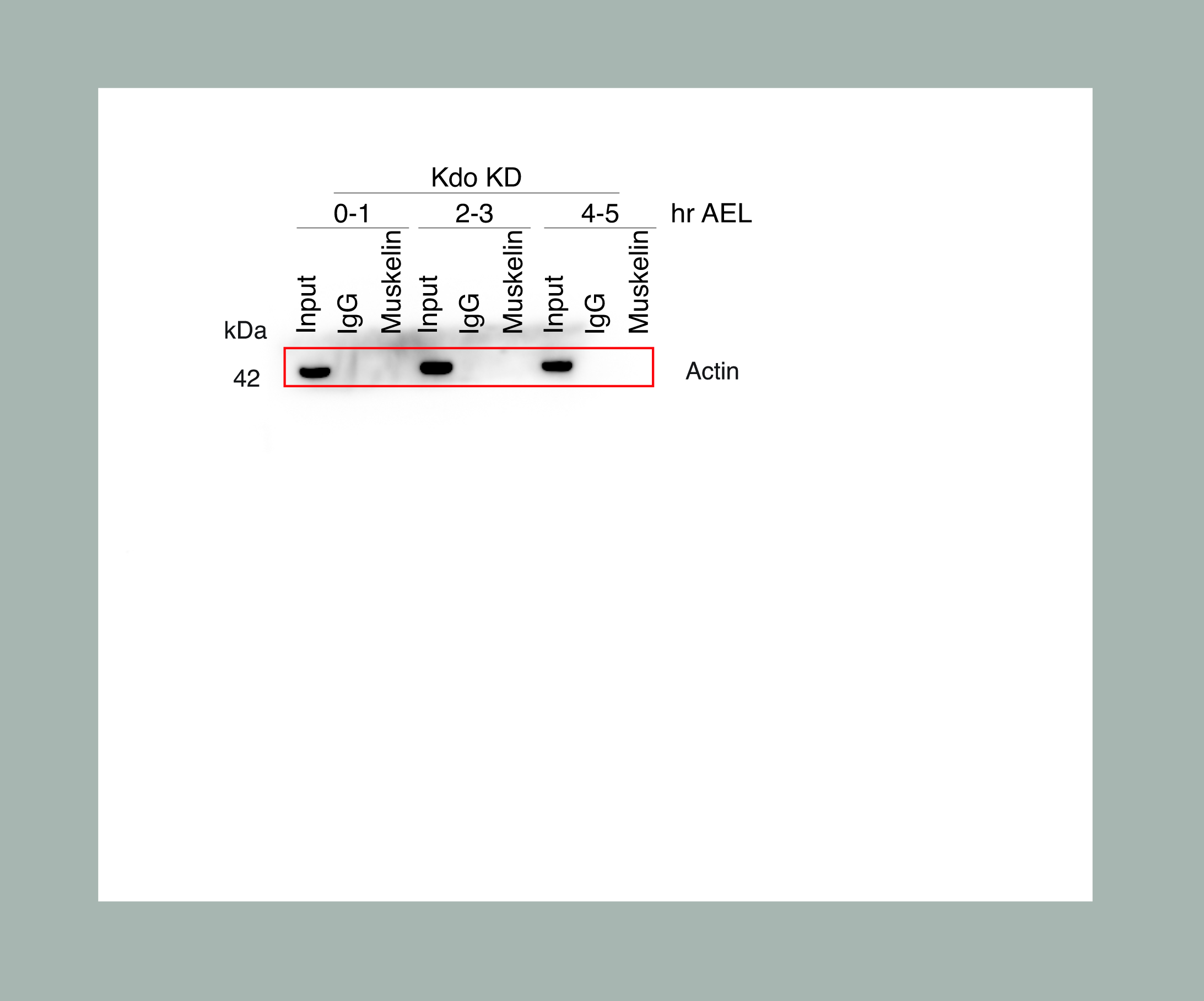

Supplement: Supplementary file 9 — Source data Fig. 1 [file 44319_2025_397_MOESM9_ESM.zip › Figure 1/1E/western actin kdokd.tif]

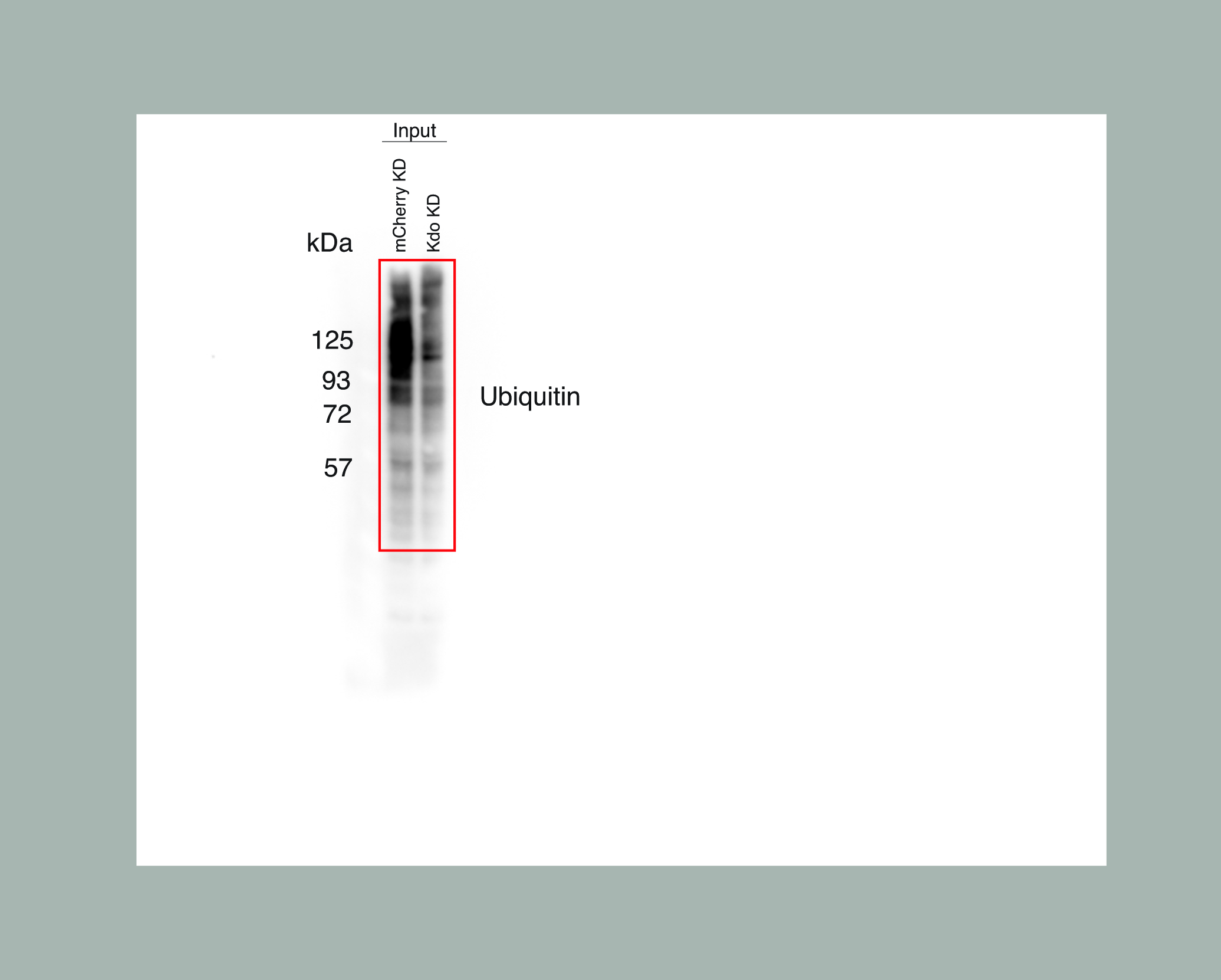

Supplement: Supplementary file 9 — Source data Fig. 1 [file 44319_2025_397_MOESM9_ESM.zip › Figure 1/1D/western ubiquitin IP.tif]

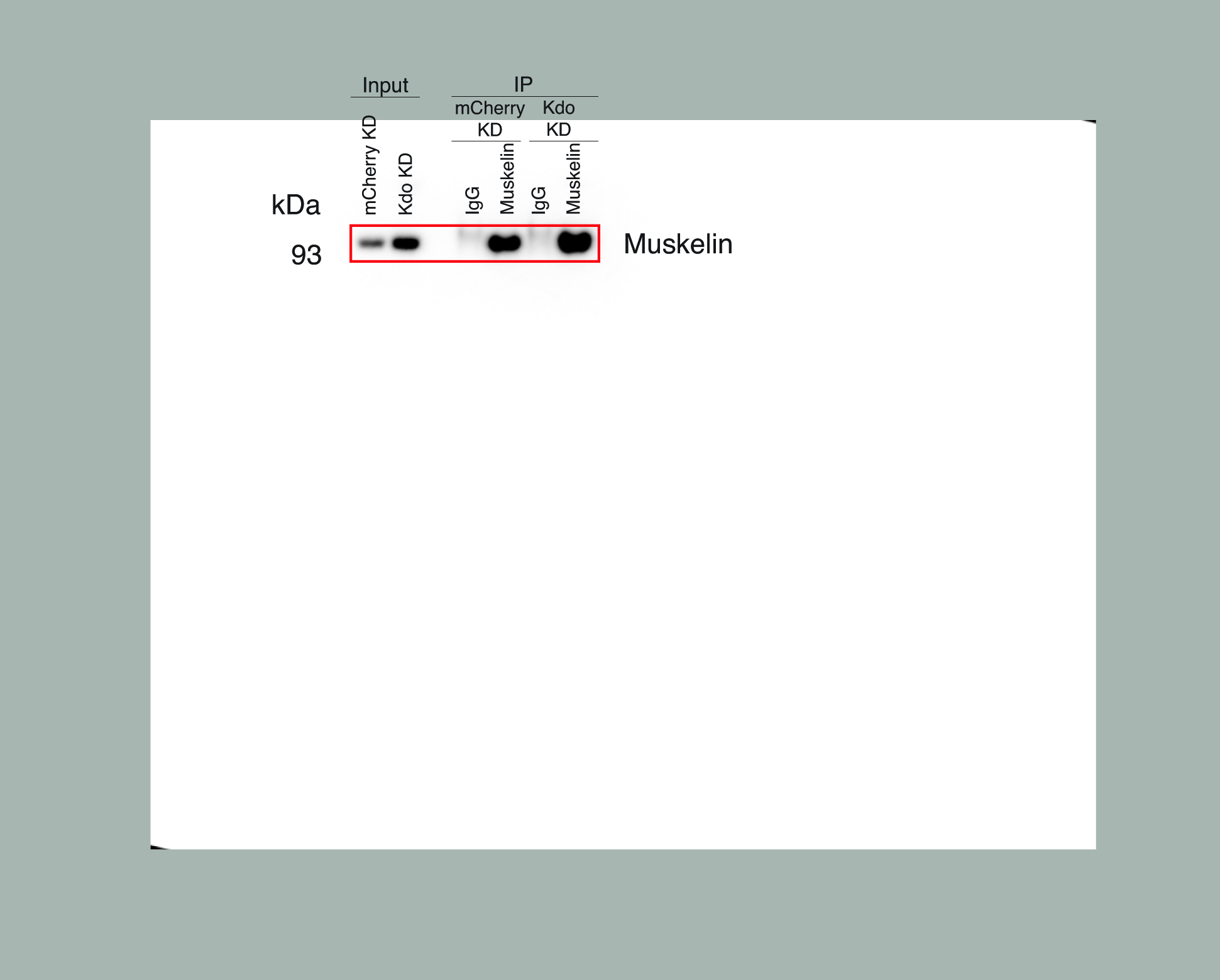

Supplement: Supplementary file 9 — Source data Fig. 1 [file 44319_2025_397_MOESM9_ESM.zip › Figure 1/1D/western muskelin.tif]

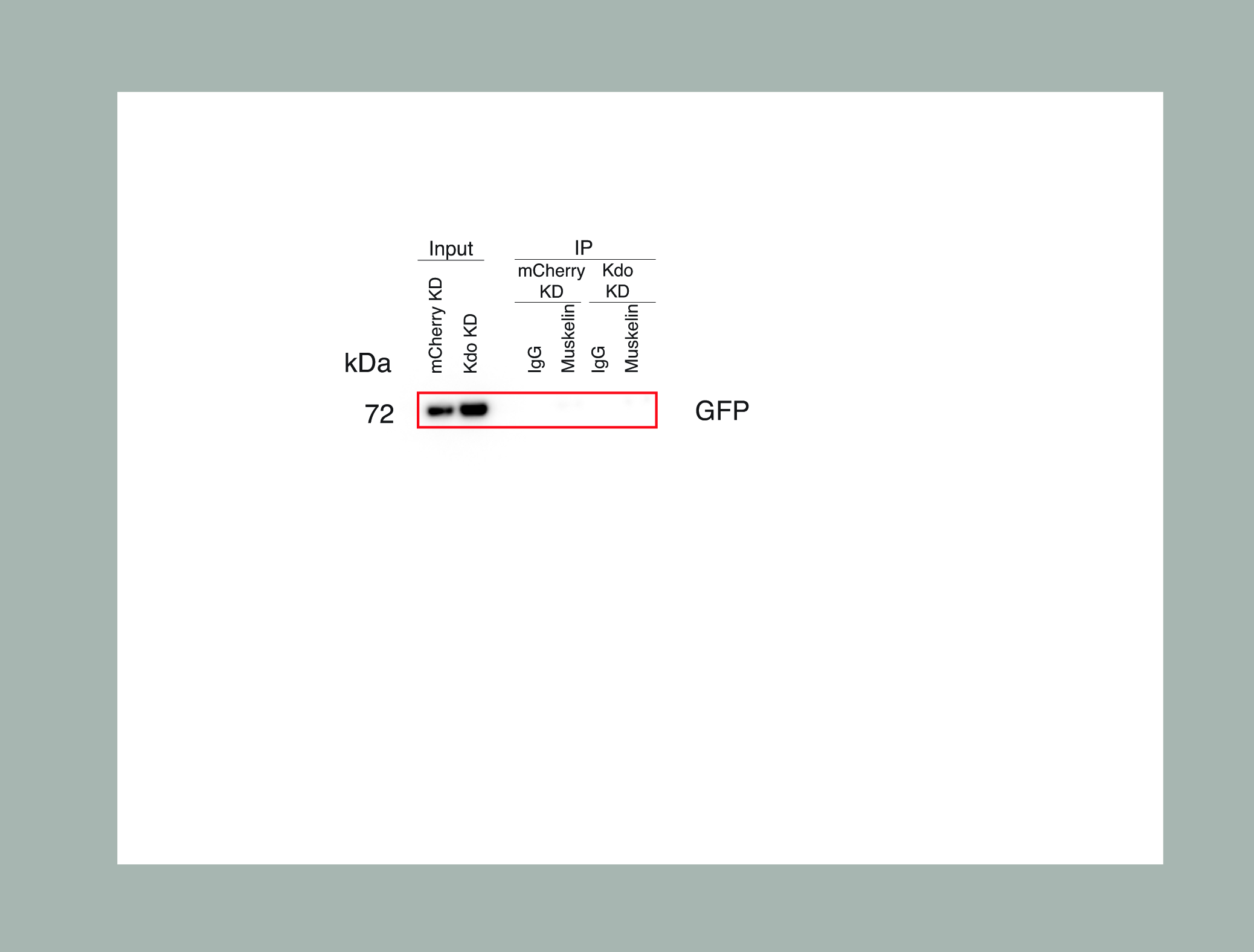

Supplement: Supplementary file 9 — Source data Fig. 1 [file 44319_2025_397_MOESM9_ESM.zip › Figure 1/1D/western gfp.tif]

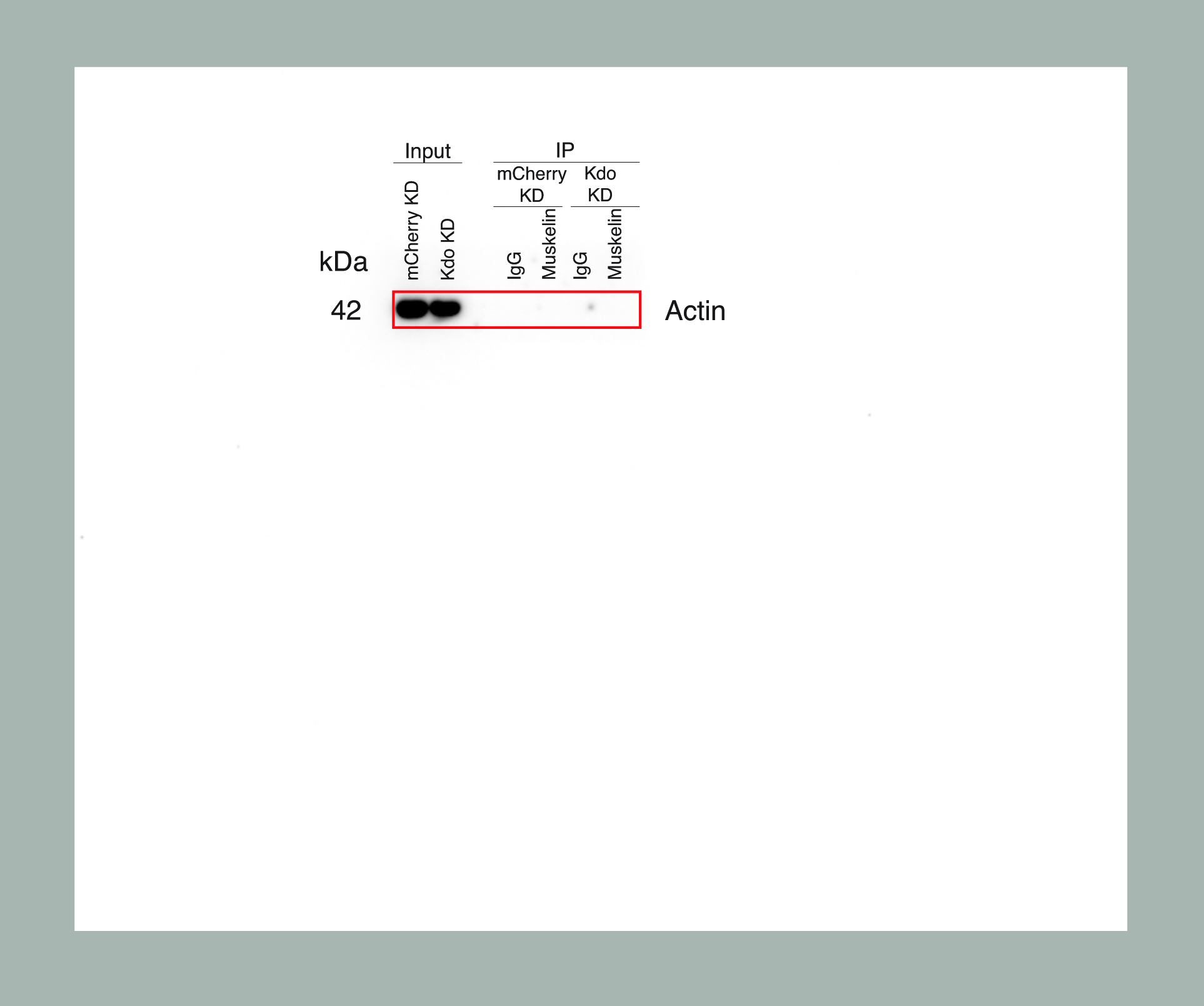

Supplement: Supplementary file 9 — Source data Fig. 1 [file 44319_2025_397_MOESM9_ESM.zip › Figure 1/1D/western actin.tif]

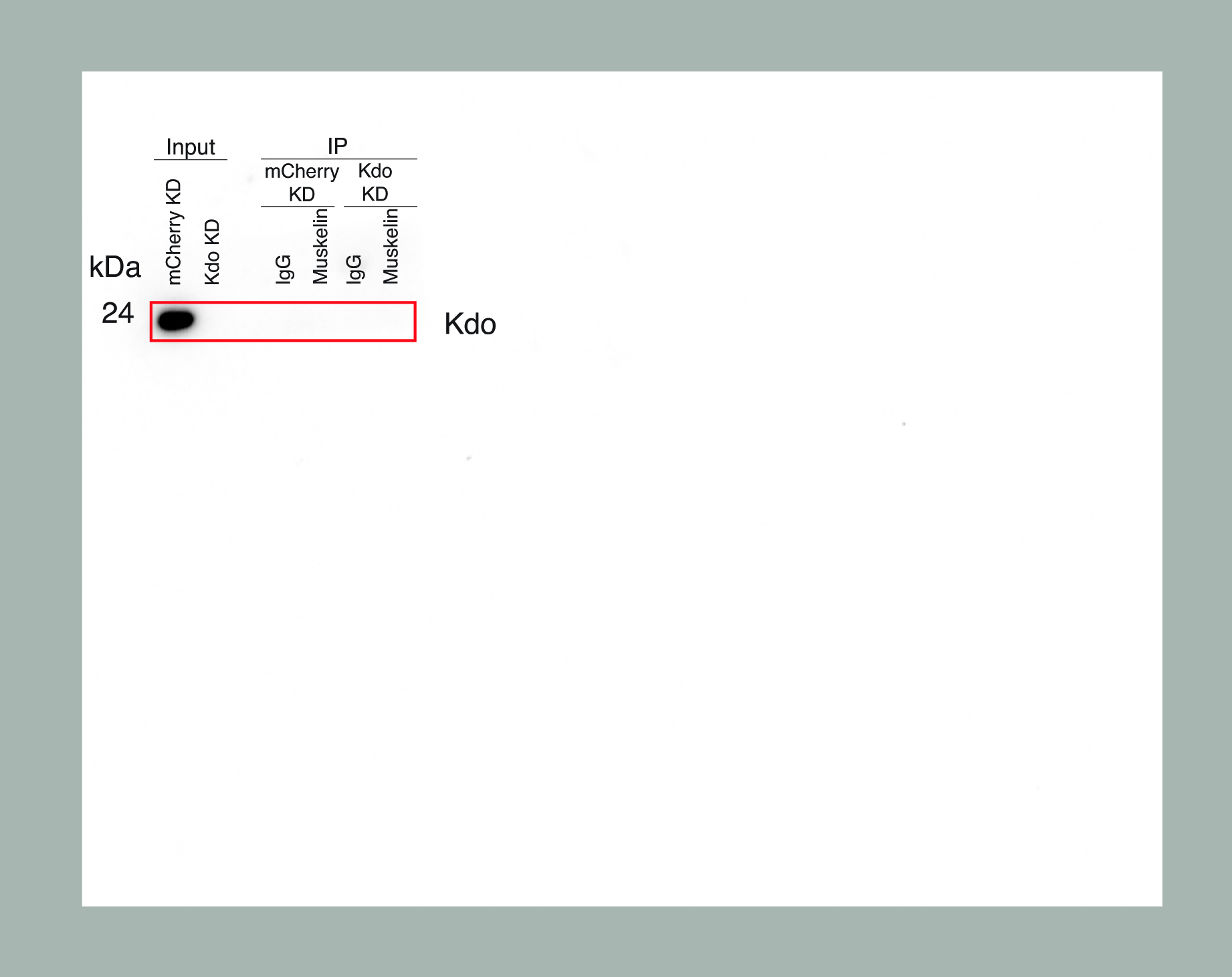

Supplement: Supplementary file 9 — Source data Fig. 1 [file 44319_2025_397_MOESM9_ESM.zip › Figure 1/1D/western kdo.tif]

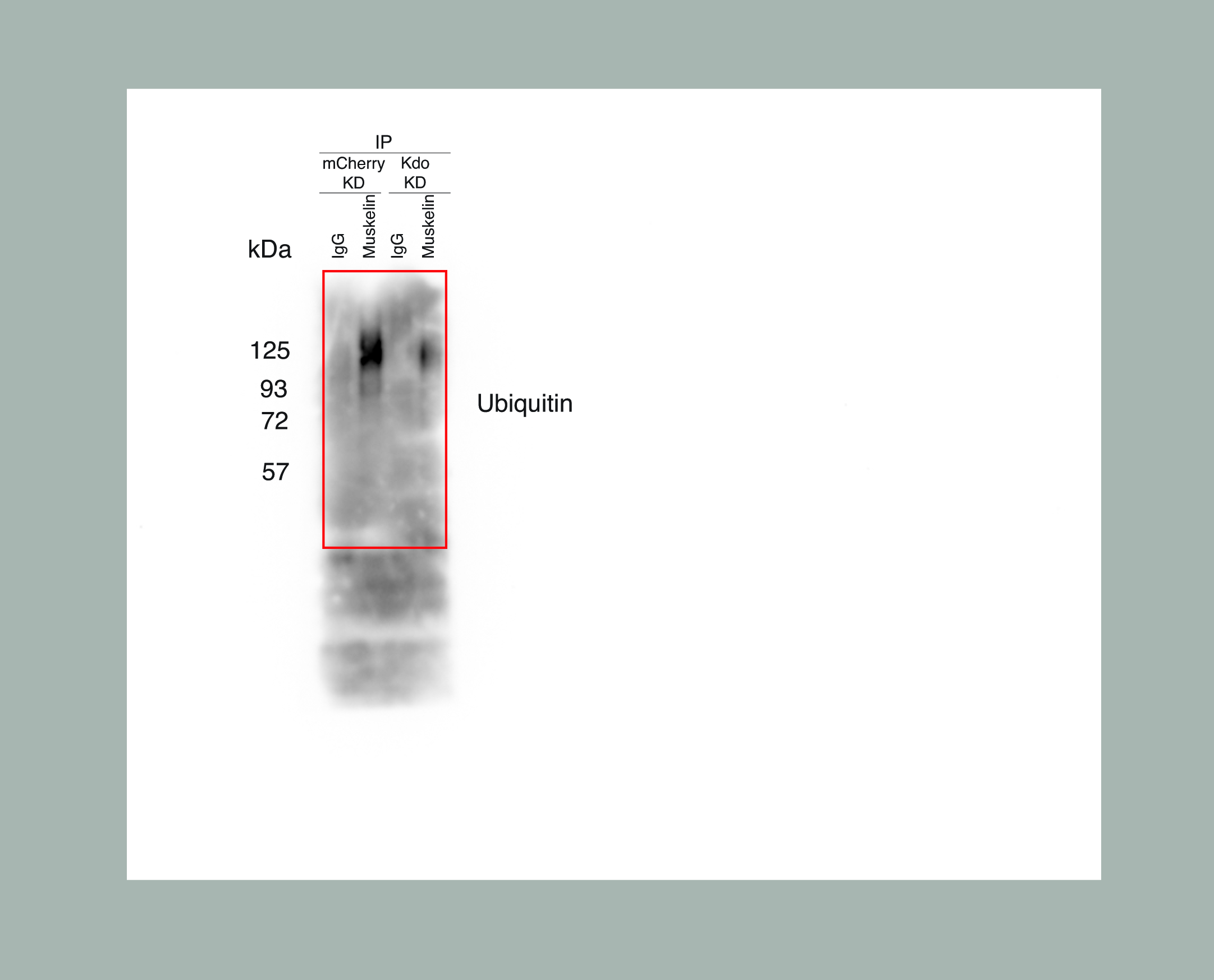

Supplement: Supplementary file 9 — Source data Fig. 1 [file 44319_2025_397_MOESM9_ESM.zip › Figure 1/1D/western ubiquitin input.tif]

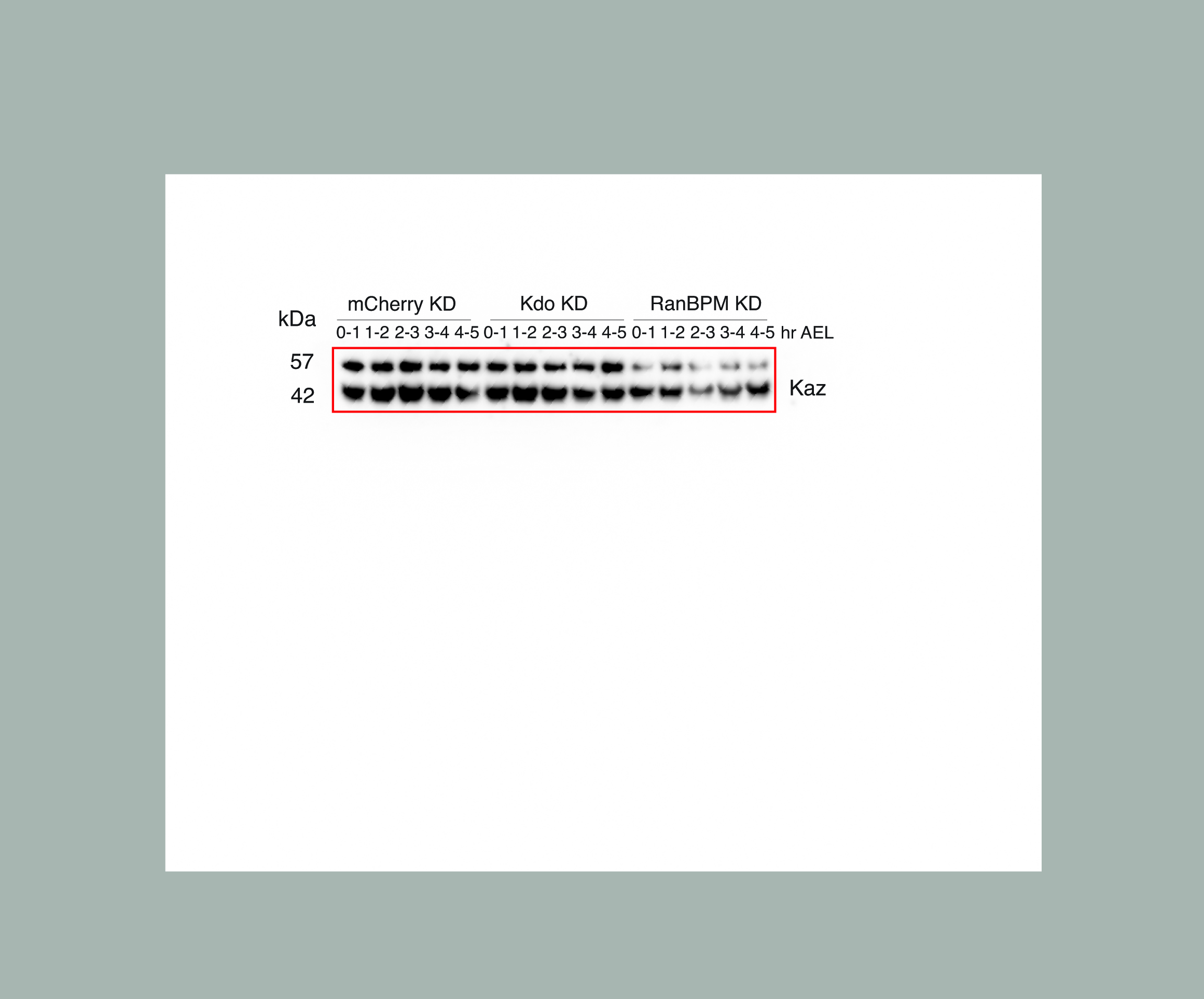

Supplement: Supplementary file 9 — Source data Fig. 1 [file 44319_2025_397_MOESM9_ESM.zip › Figure 1/1C/western kaz.tif]

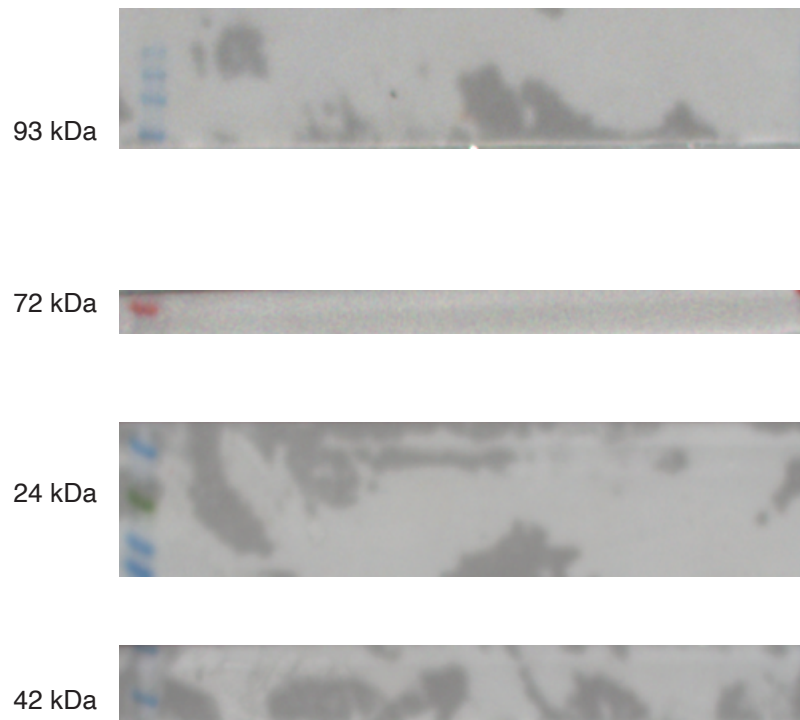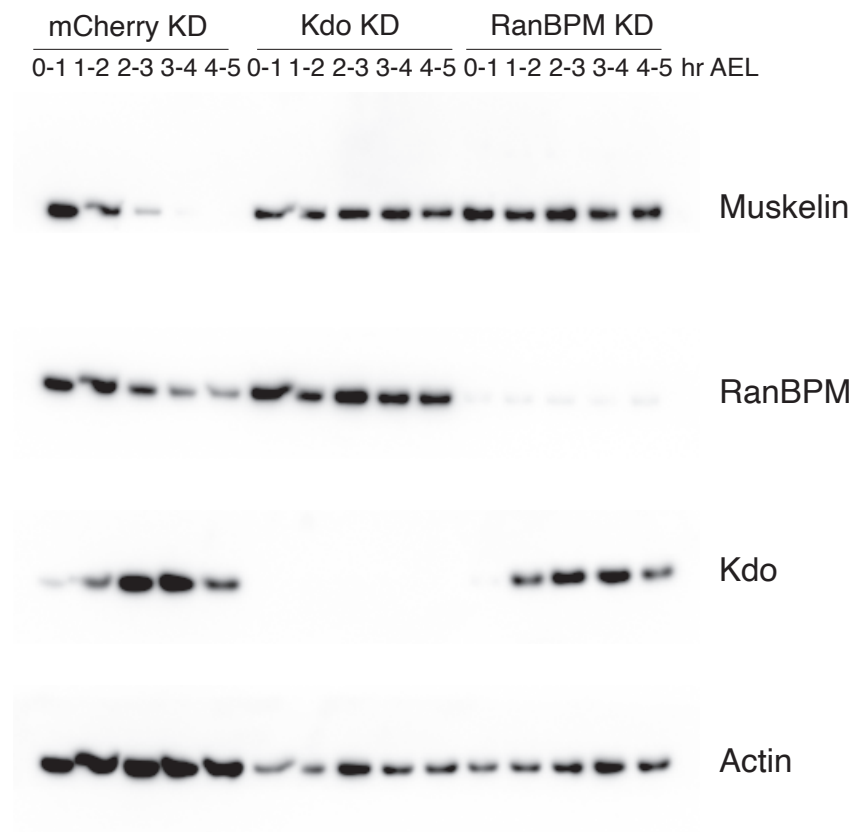

Supplement: Supplementary file 9 — Source data Fig. 1 [file 44319_2025_397_MOESM9_ESM.zip › Figure 1/1C/Fig1C_clarity.pdf]

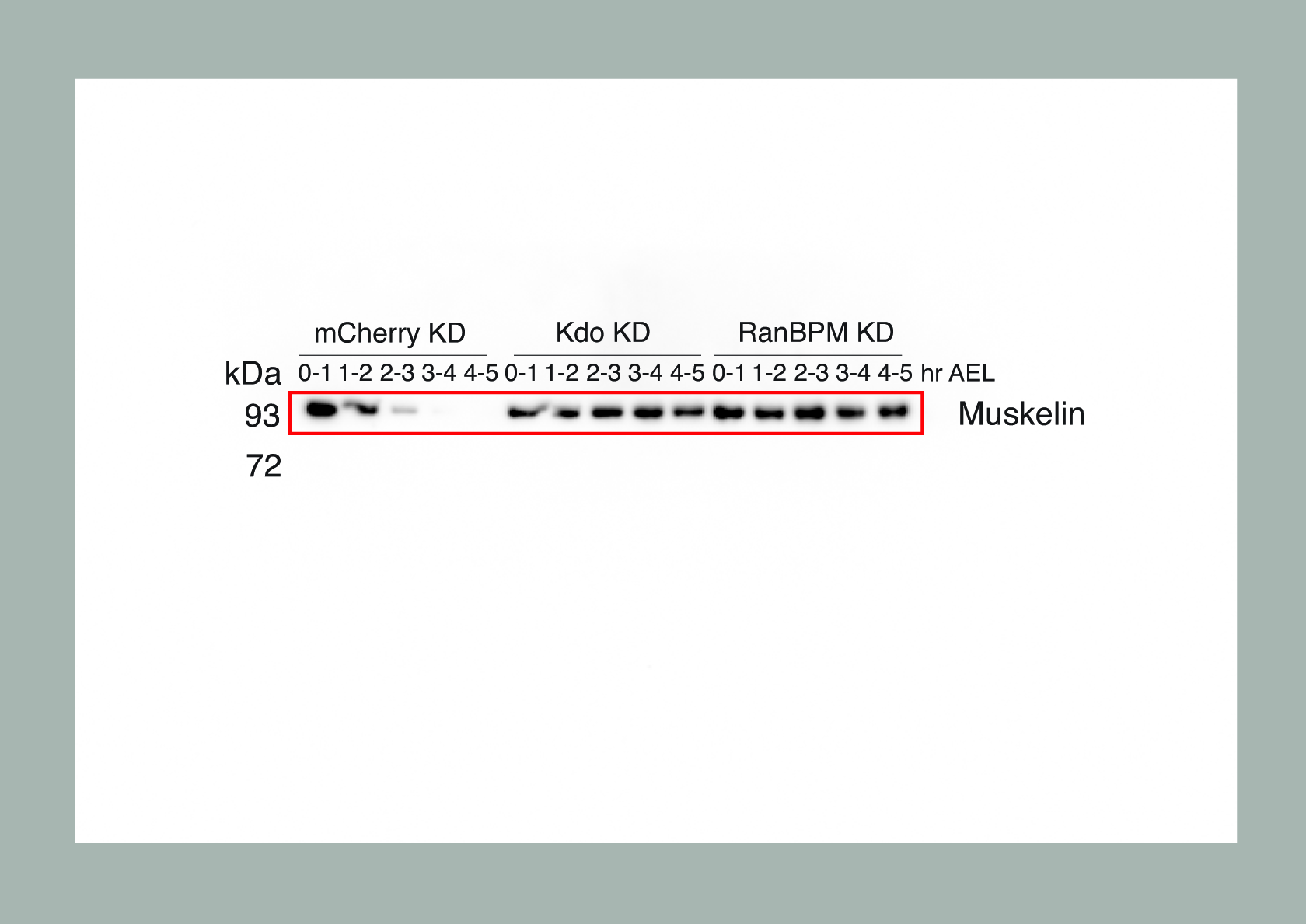

Supplement: Supplementary file 9 — Source data Fig. 1 [file 44319_2025_397_MOESM9_ESM.zip › Figure 1/1C/western muskelin.tif]

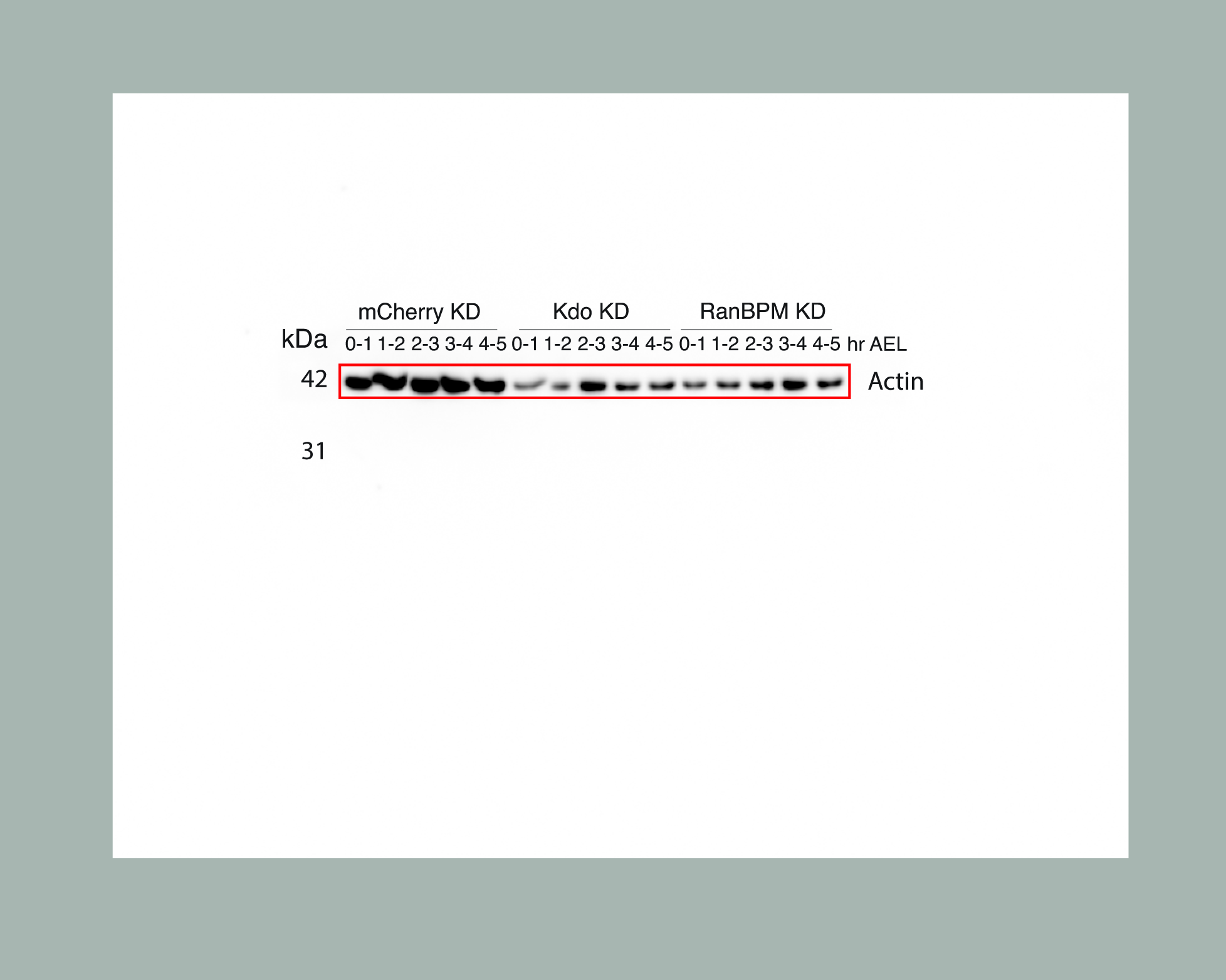

Supplement: Supplementary file 9 — Source data Fig. 1 [file 44319_2025_397_MOESM9_ESM.zip › Figure 1/1C/western actin.tif]

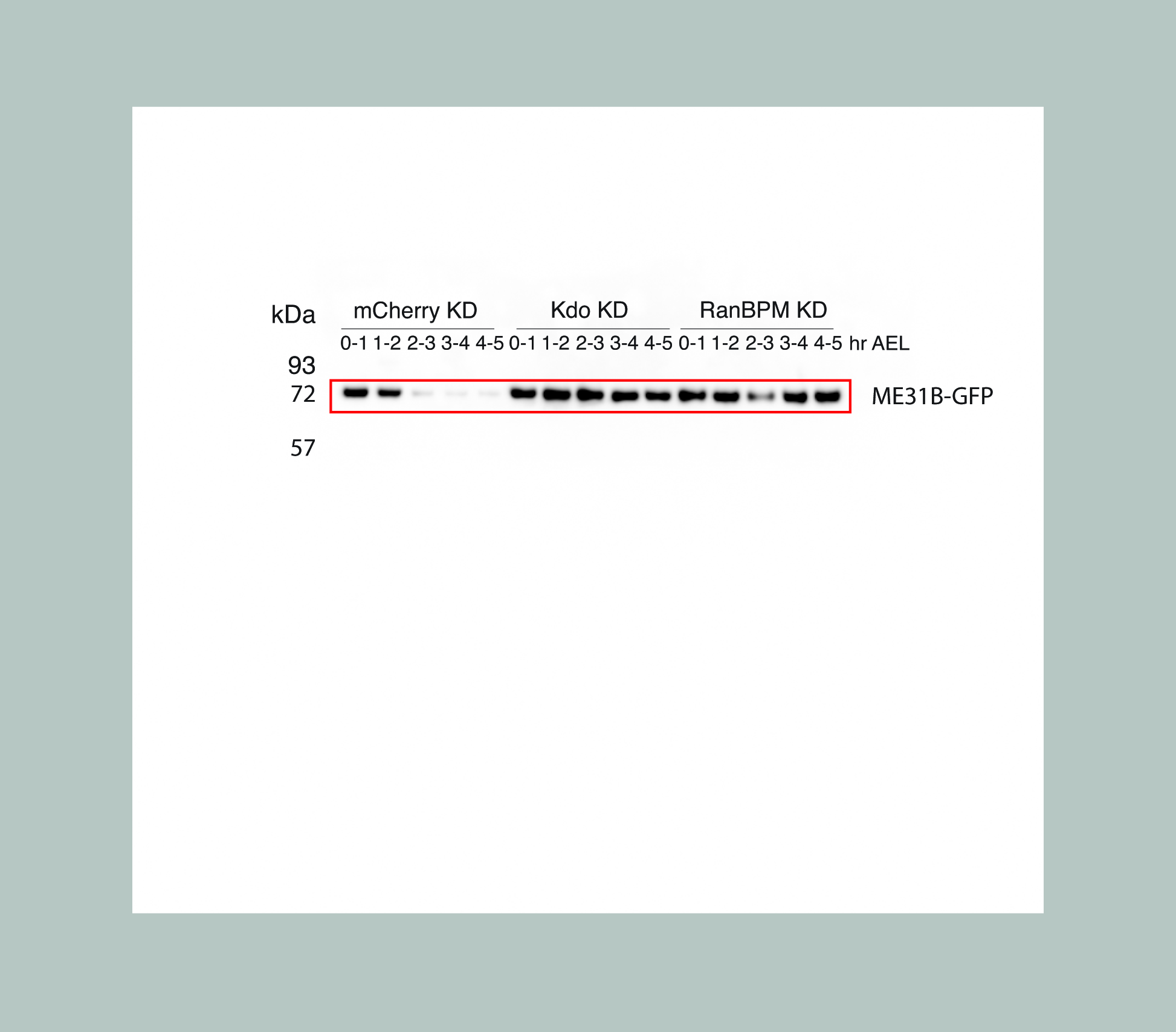

Supplement: Supplementary file 9 — Source data Fig. 1 [file 44319_2025_397_MOESM9_ESM.zip › Figure 1/1C/western me31b-gfp.tif]

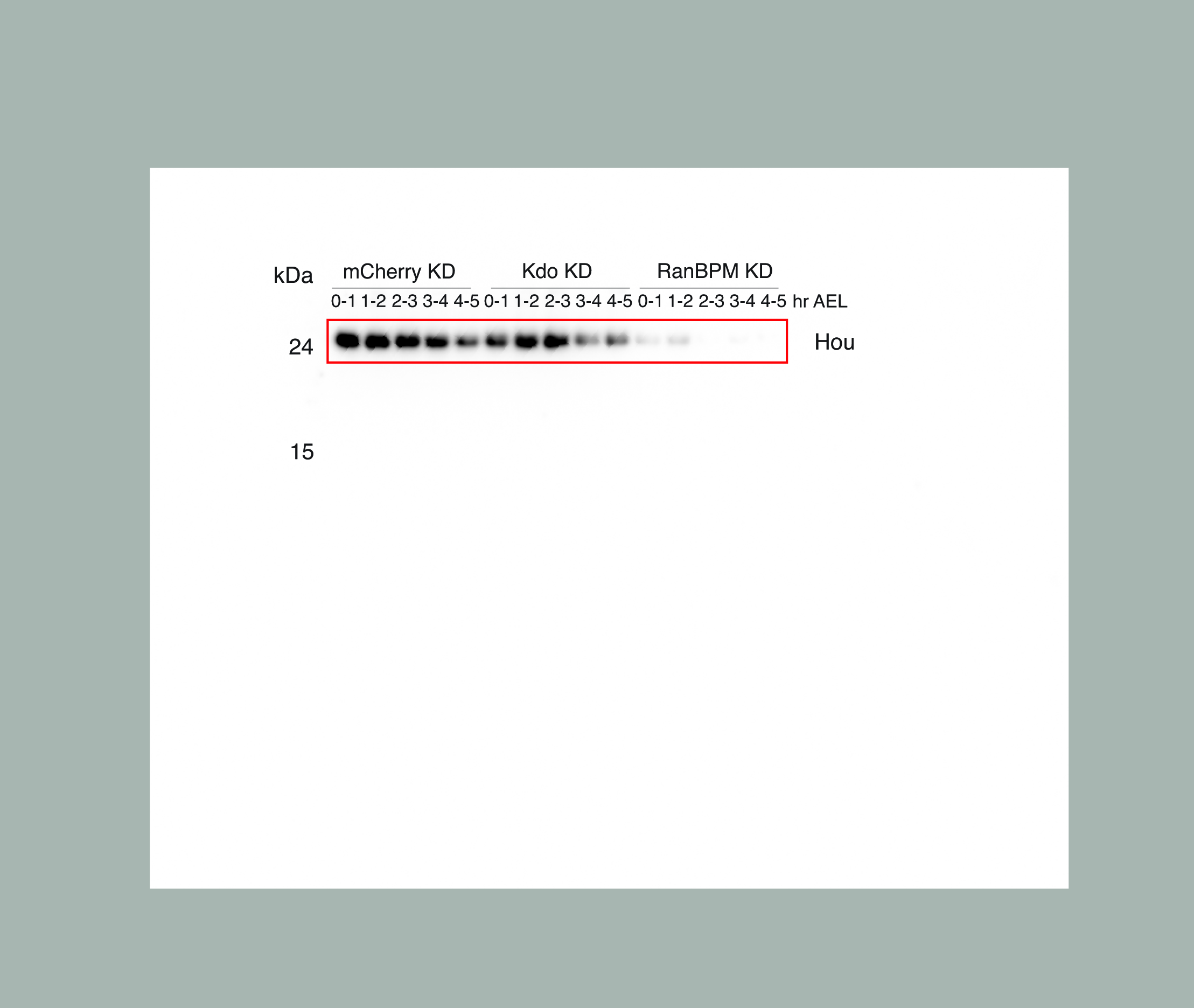

Supplement: Supplementary file 9 — Source data Fig. 1 [file 44319_2025_397_MOESM9_ESM.zip › Figure 1/1C/western hou.tif]

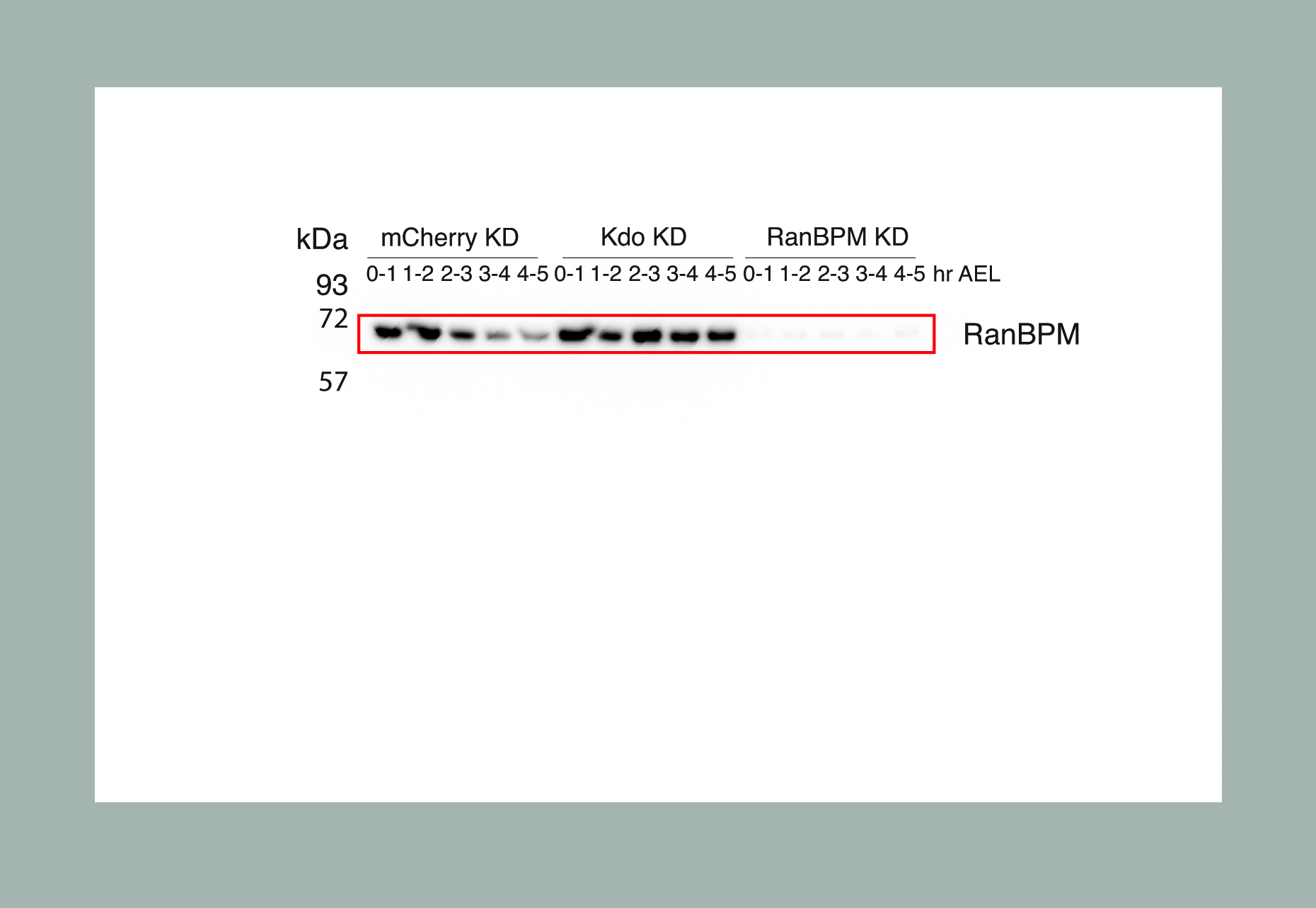

Supplement: Supplementary file 9 — Source data Fig. 1 [file 44319_2025_397_MOESM9_ESM.zip › Figure 1/1C/western ranbpm.tif]

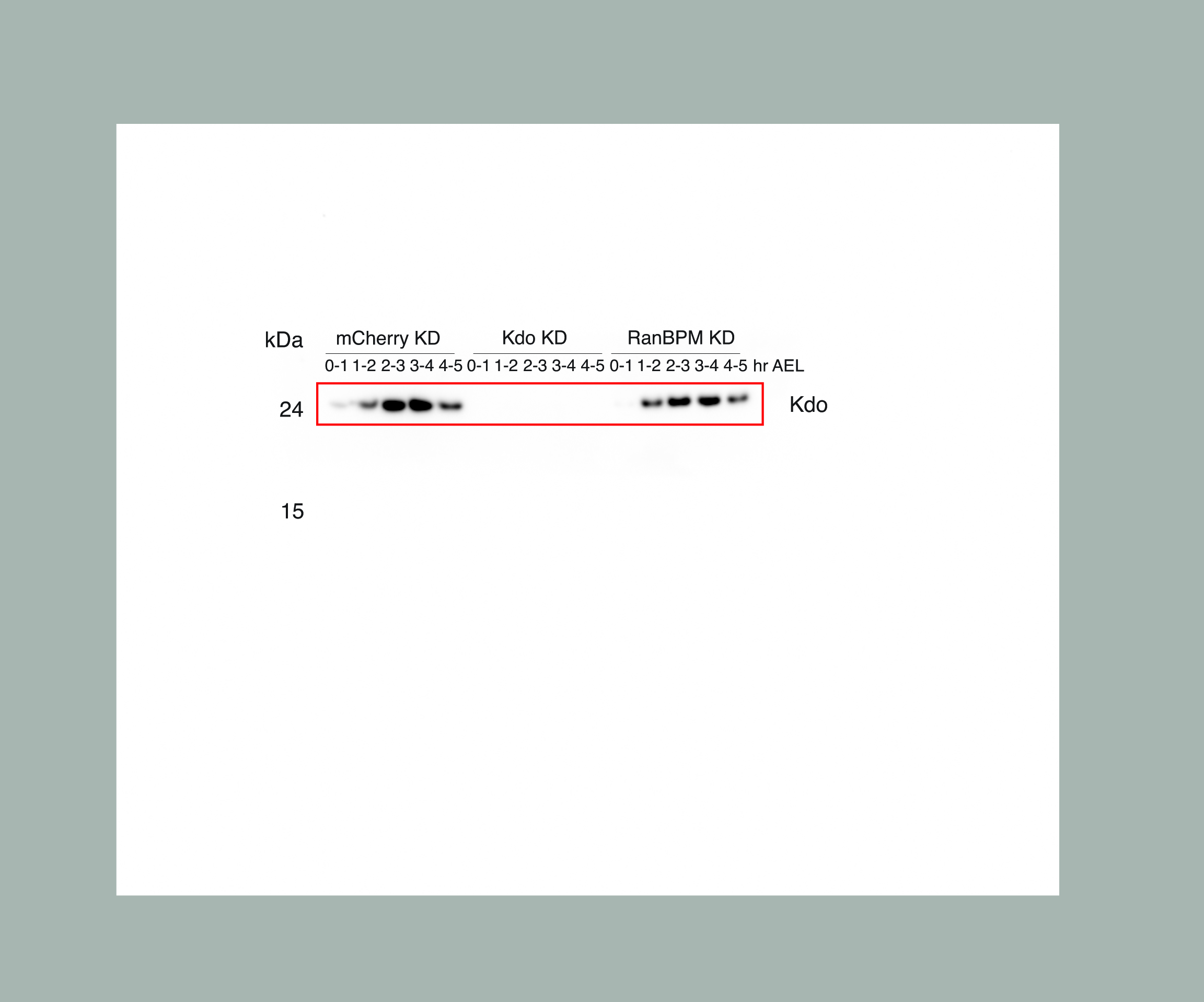

Supplement: Supplementary file 9 — Source data Fig. 1 [file 44319_2025_397_MOESM9_ESM.zip › Figure 1/1C/western kdo.tif]

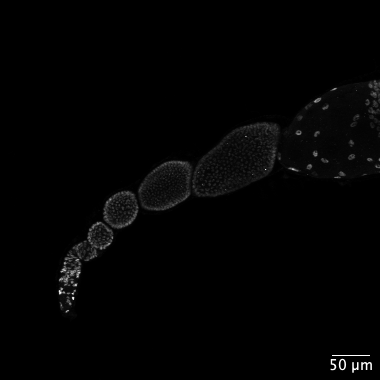

Supplement: Supplementary file 10 — Source data Fig. 2 [file 44319_2025_397_MOESM10_ESM.zip › Figure 2/C/MAX_tj594_2C'''.tif]

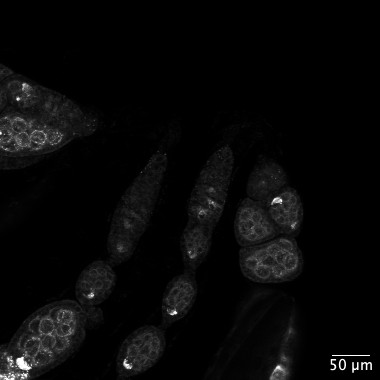

Supplement: Supplementary file 10 — Source data Fig. 2 [file 44319_2025_397_MOESM10_ESM.zip › Figure 2/C/MAX_bam-GFPonly_2C'.tif]

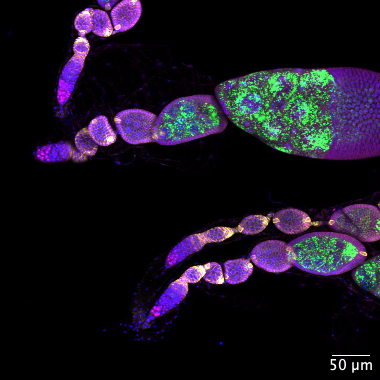

Supplement: Supplementary file 10 — Source data Fig. 2 [file 44319_2025_397_MOESM10_ESM.zip › Figure 2/C/MAX_fas3-568_musk647_1_GFP_2C''.tif]

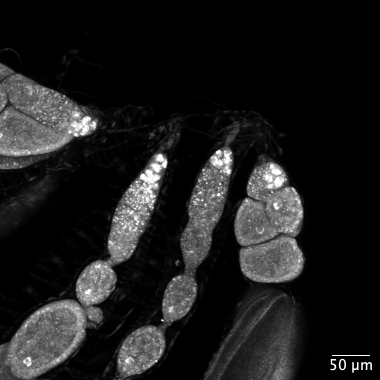

Supplement: Supplementary file 10 — Source data Fig. 2 [file 44319_2025_397_MOESM10_ESM.zip › Figure 2/C/MAX_bam-musk647_2C'.tif]

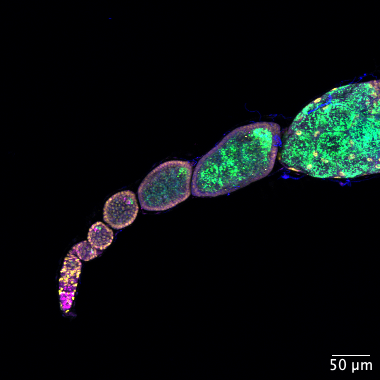

Supplement: Supplementary file 10 — Source data Fig. 2 [file 44319_2025_397_MOESM10_ESM.zip › Figure 2/C/MAX_tj594_musk647_GFP_2C'''.tif]

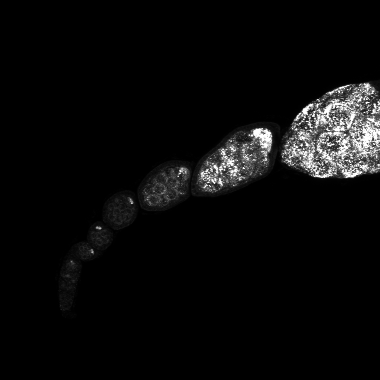

Supplement: Supplementary file 10 — Source data Fig. 2 [file 44319_2025_397_MOESM10_ESM.zip › Figure 2/C/MAX_tj-GFPonly_2C'''.tif]

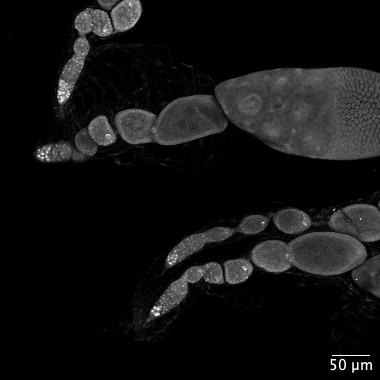

Supplement: Supplementary file 10 — Source data Fig. 2 [file 44319_2025_397_MOESM10_ESM.zip › Figure 2/C/MAX_fas3-musk647_2C''.tif]

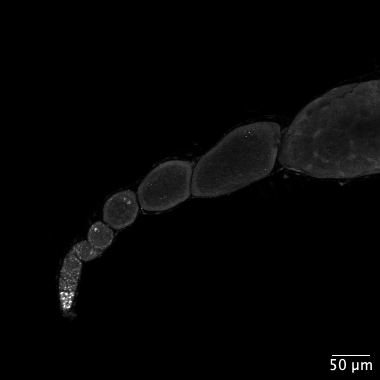

Supplement: Supplementary file 10 — Source data Fig. 2 [file 44319_2025_397_MOESM10_ESM.zip › Figure 2/C/MAX_tj-musk647_2C'''.tif]

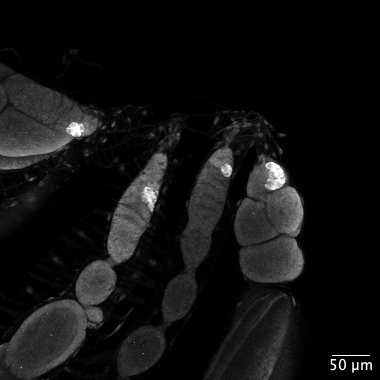

Supplement: Supplementary file 10 — Source data Fig. 2 [file 44319_2025_397_MOESM10_ESM.zip › Figure 2/C/MAX_bam568_2C'.tif]

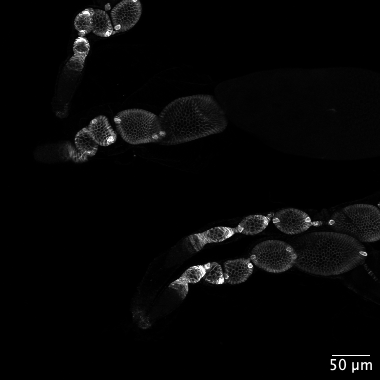

Supplement: Supplementary file 10 — Source data Fig. 2 [file 44319_2025_397_MOESM10_ESM.zip › Figure 2/C/MAX_fas3-568_2C''.tif]

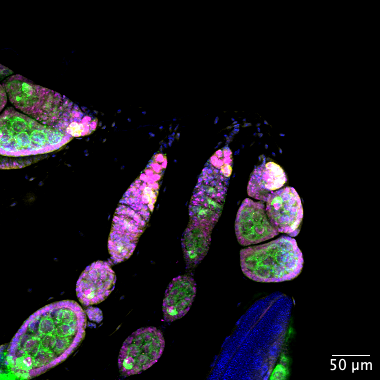

Supplement: Supplementary file 10 — Source data Fig. 2 [file 44319_2025_397_MOESM10_ESM.zip › Figure 2/C/MAX_bam568_musk647_GFP_2C'.tif]

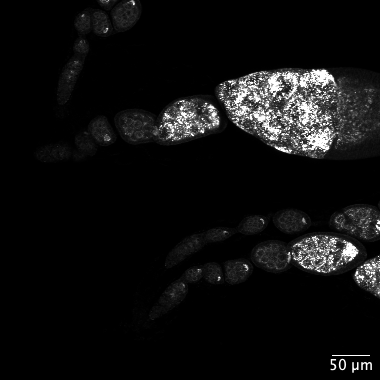

Supplement: Supplementary file 10 — Source data Fig. 2 [file 44319_2025_397_MOESM10_ESM.zip › Figure 2/C/MAX_fas3-GFPonly_2C''.tif]

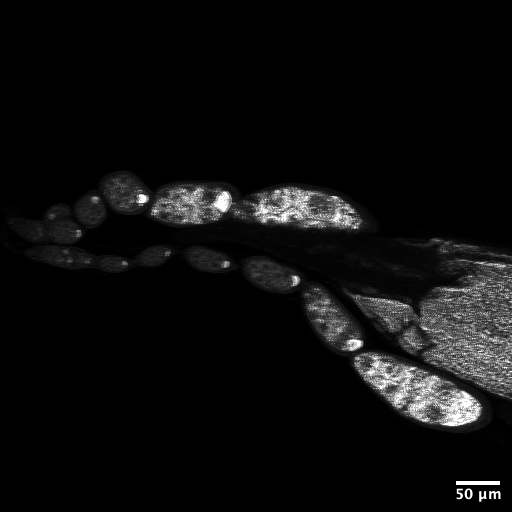

Supplement: Supplementary file 10 — Source data Fig. 2 [file 44319_2025_397_MOESM10_ESM.zip › Figure 2/D/MAX_WT_GFPonly_2D.tif]

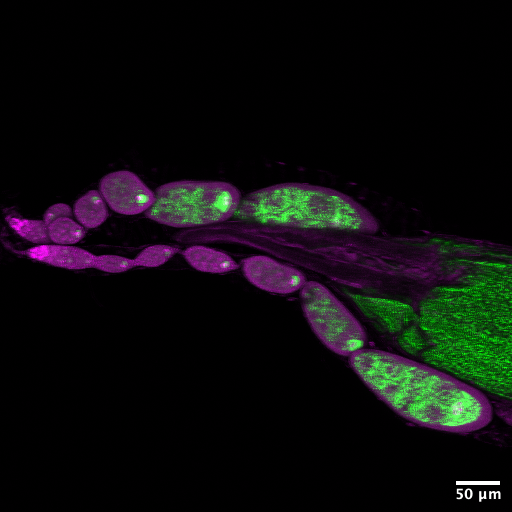

Supplement: Supplementary file 10 — Source data Fig. 2 [file 44319_2025_397_MOESM10_ESM.zip › Figure 2/D/MAX_WT_Musk_GFP_flat_2D.tif]

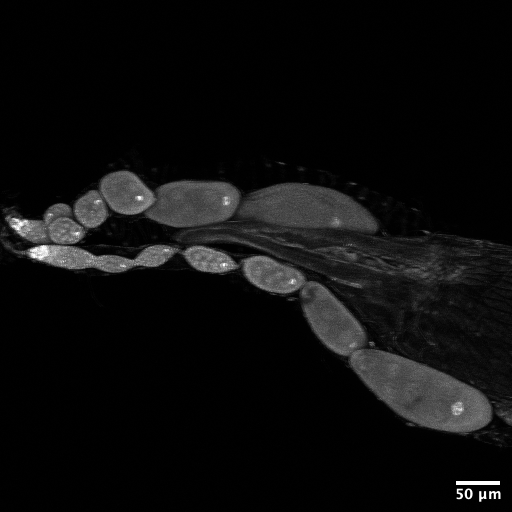

Supplement: Supplementary file 10 — Source data Fig. 2 [file 44319_2025_397_MOESM10_ESM.zip › Figure 2/D/MAX_WT_Muskonly_2D.tif]

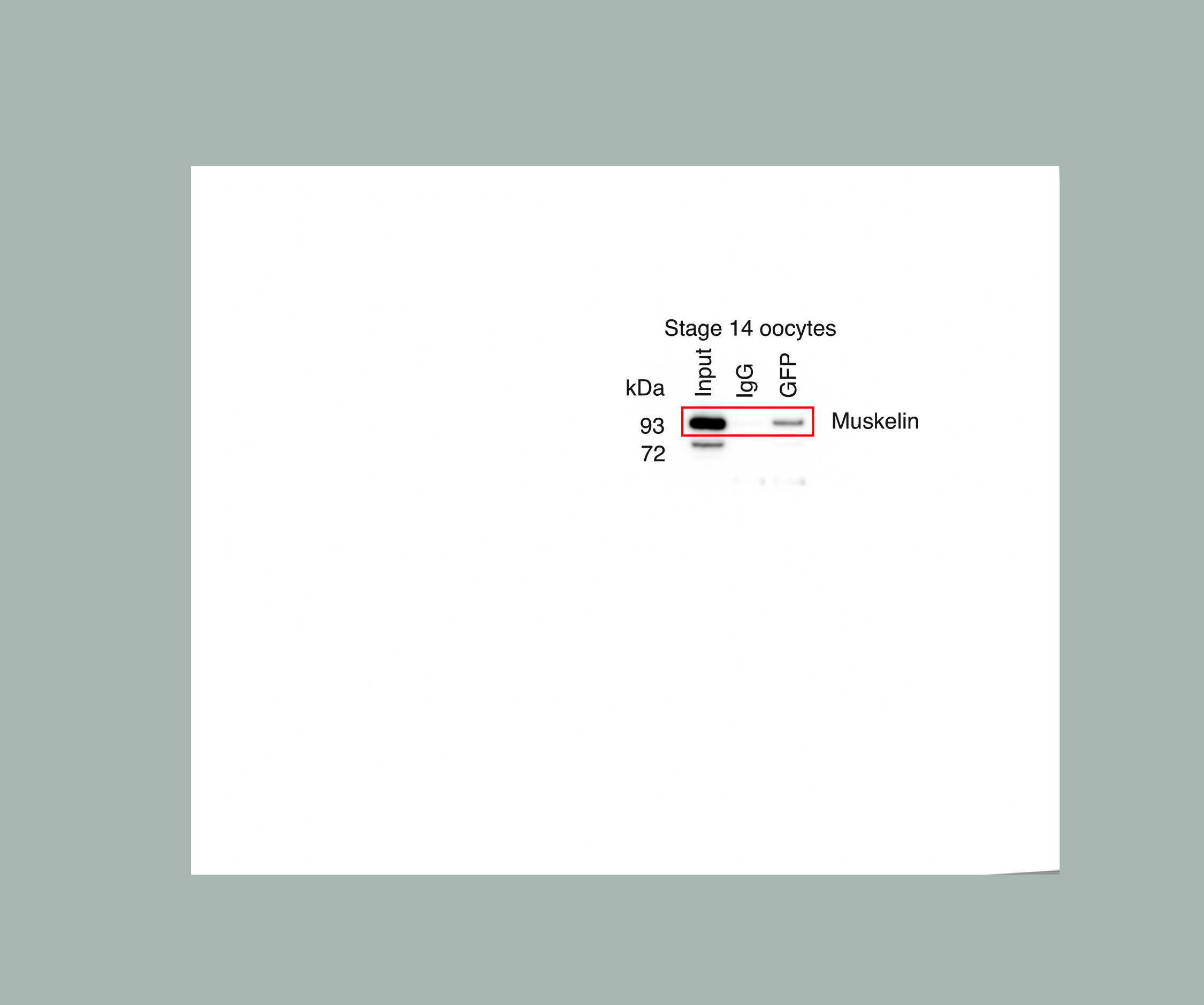

Supplement: Supplementary file 10 — Source data Fig. 2 [file 44319_2025_397_MOESM10_ESM.zip › Figure 2/E/western muskelin.tif]

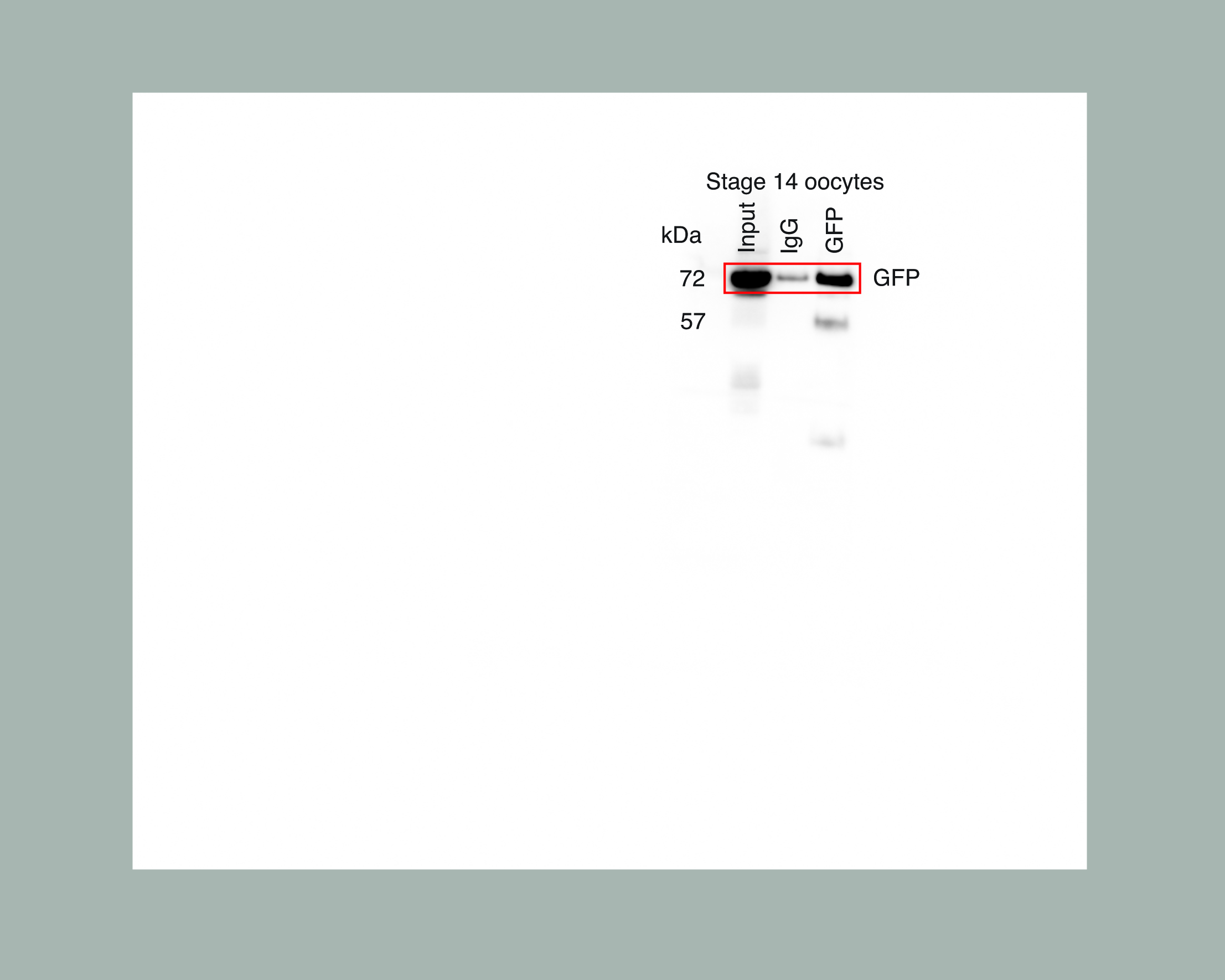

Supplement: Supplementary file 10 — Source data Fig. 2 [file 44319_2025_397_MOESM10_ESM.zip › Figure 2/E/western gfp.tif]

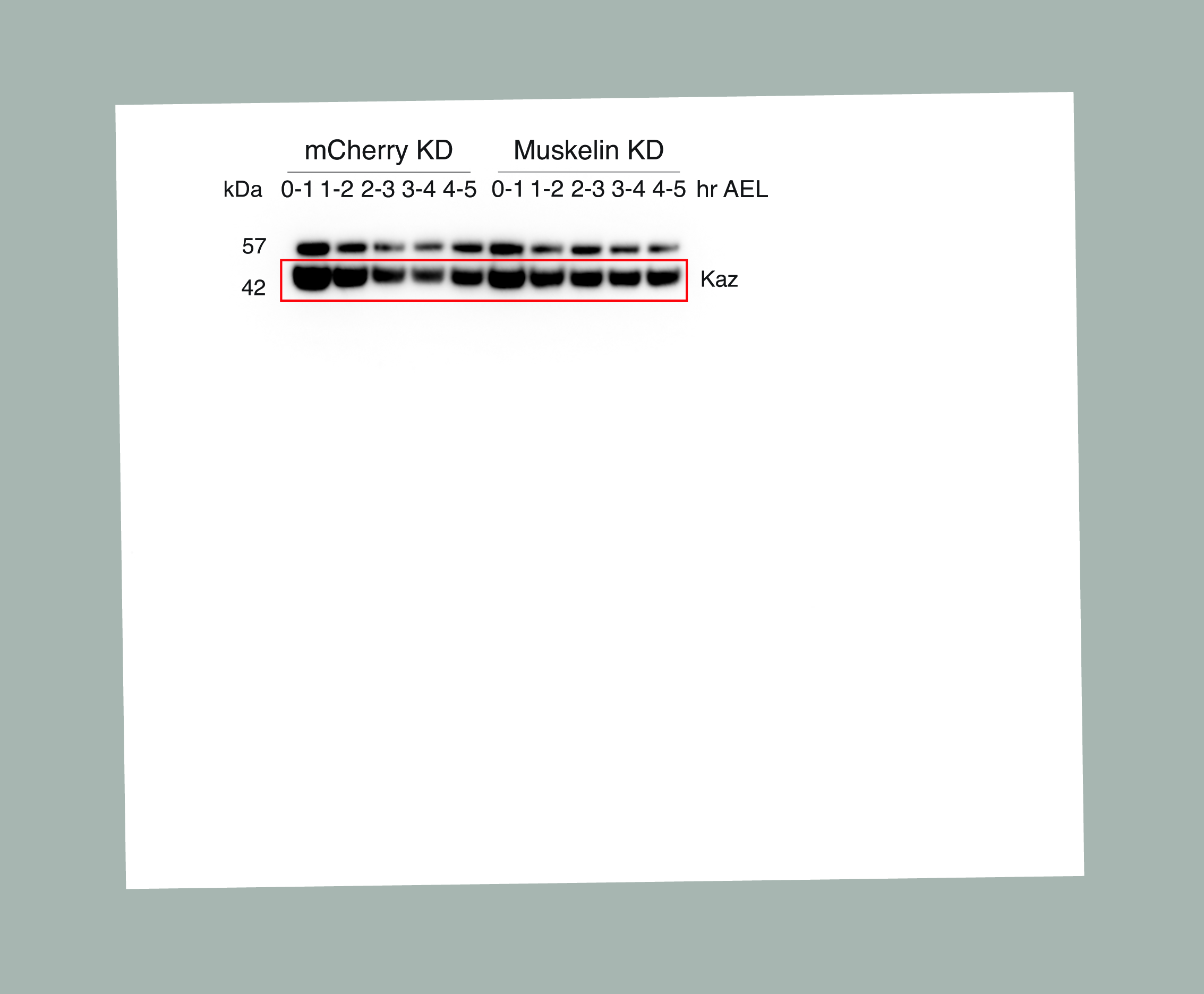

Supplement: Supplementary file 11 — Source data Fig. 3 [file 44319_2025_397_MOESM11_ESM.zip › Figure 3/A/western kaz.tif]

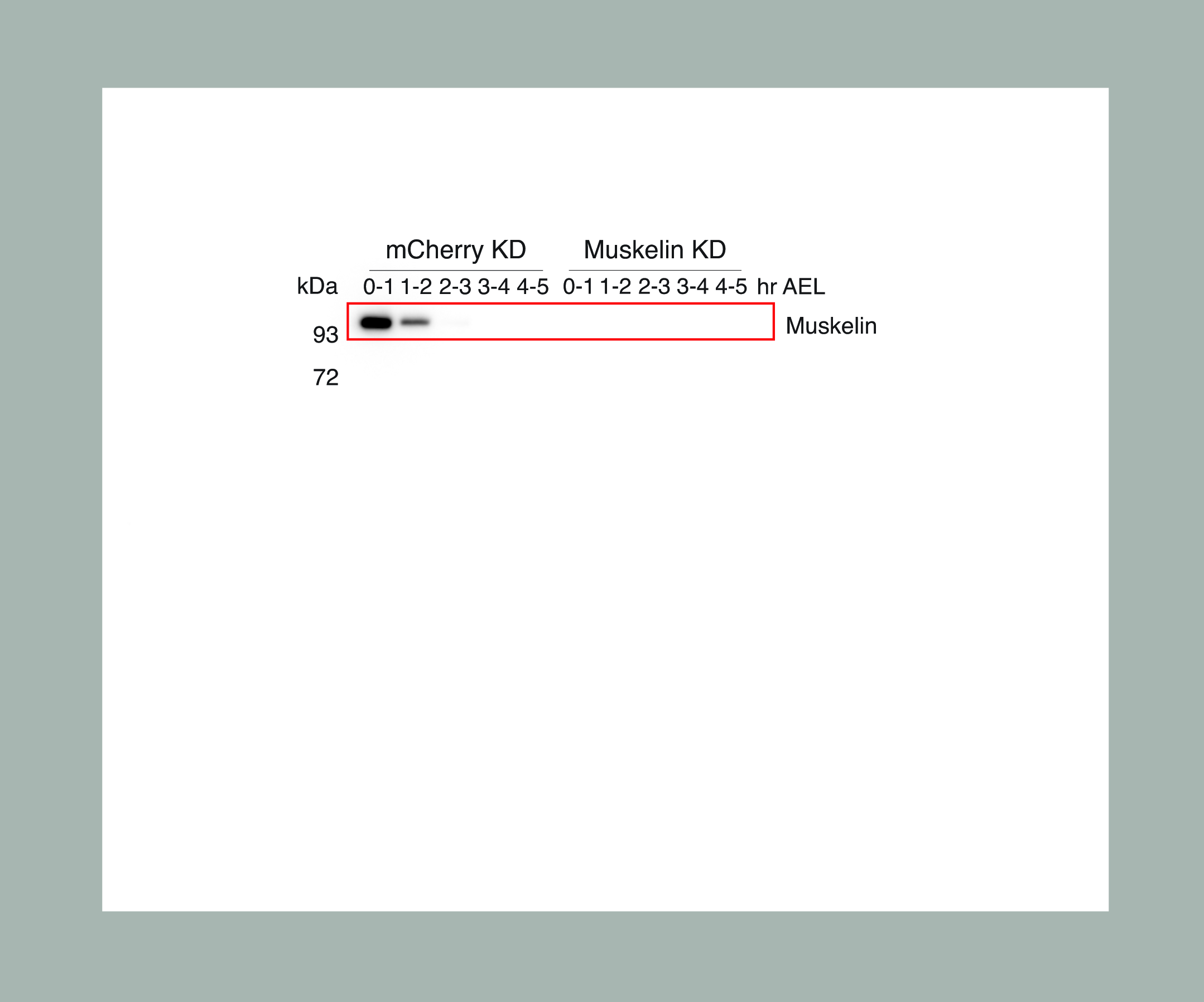

Supplement: Supplementary file 11 — Source data Fig. 3 [file 44319_2025_397_MOESM11_ESM.zip › Figure 3/A/western muskelin.tif]

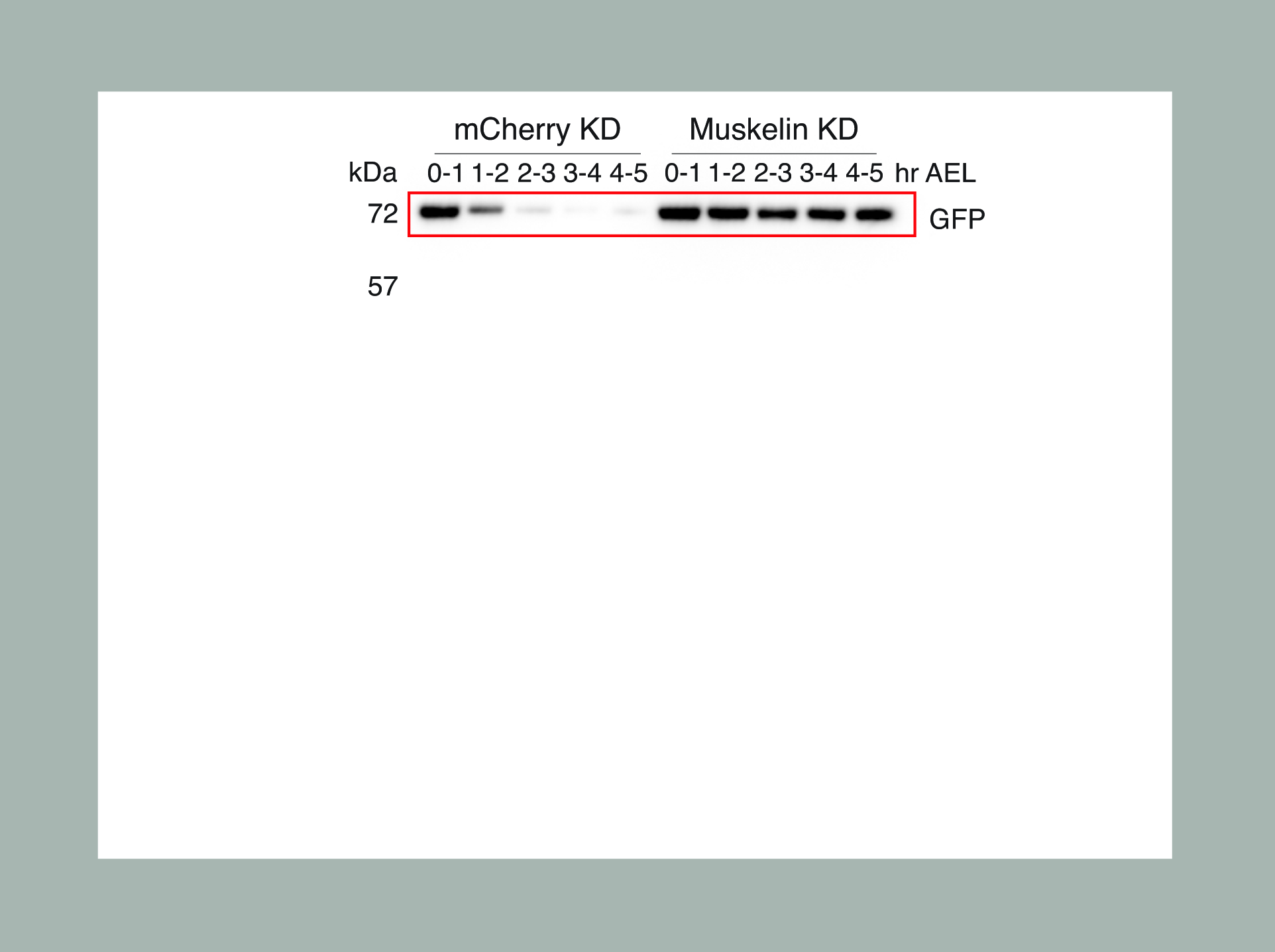

Supplement: Supplementary file 11 — Source data Fig. 3 [file 44319_2025_397_MOESM11_ESM.zip › Figure 3/A/western gfp.tif]

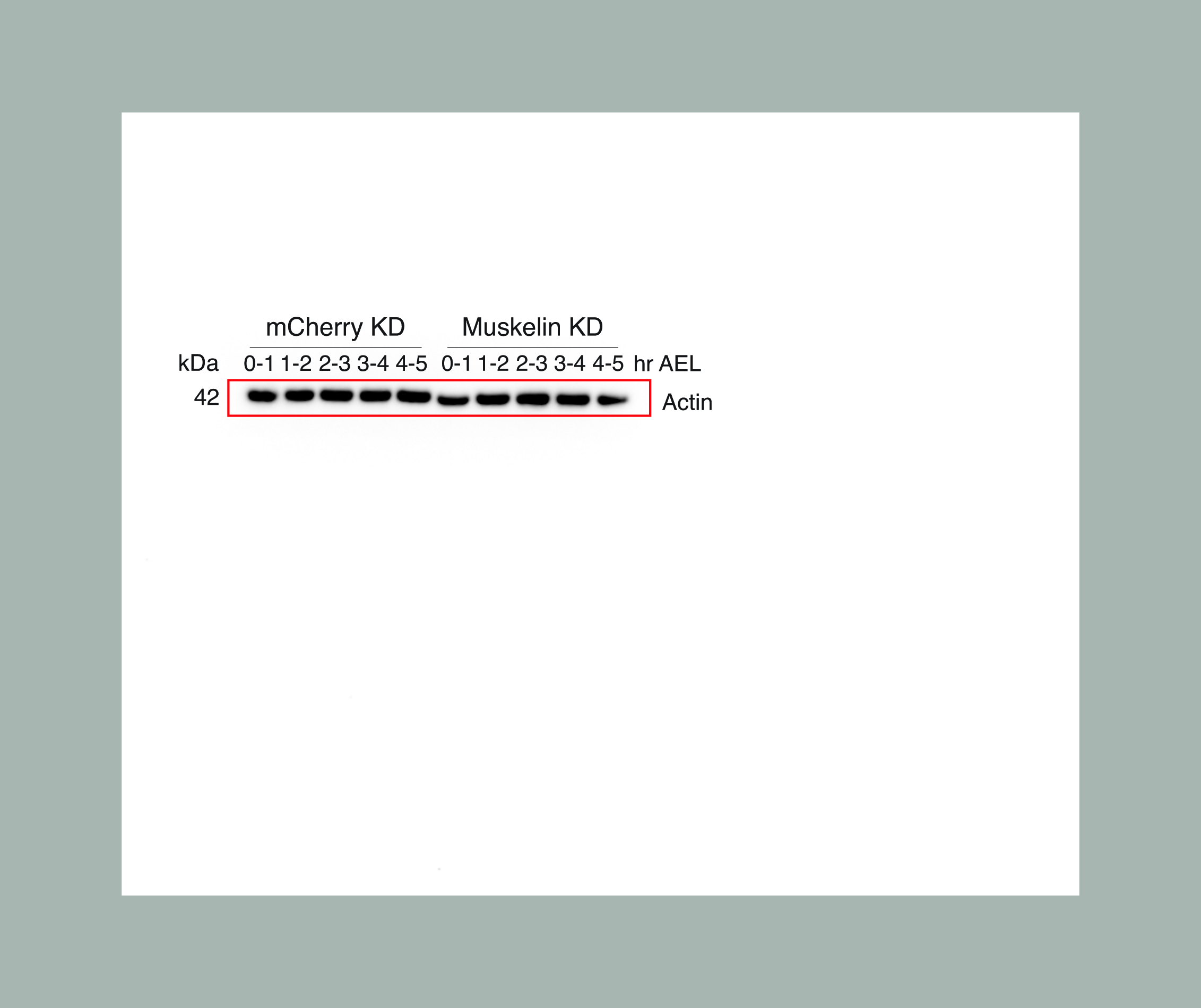

Supplement: Supplementary file 11 — Source data Fig. 3 [file 44319_2025_397_MOESM11_ESM.zip › Figure 3/A/western actin.tif]

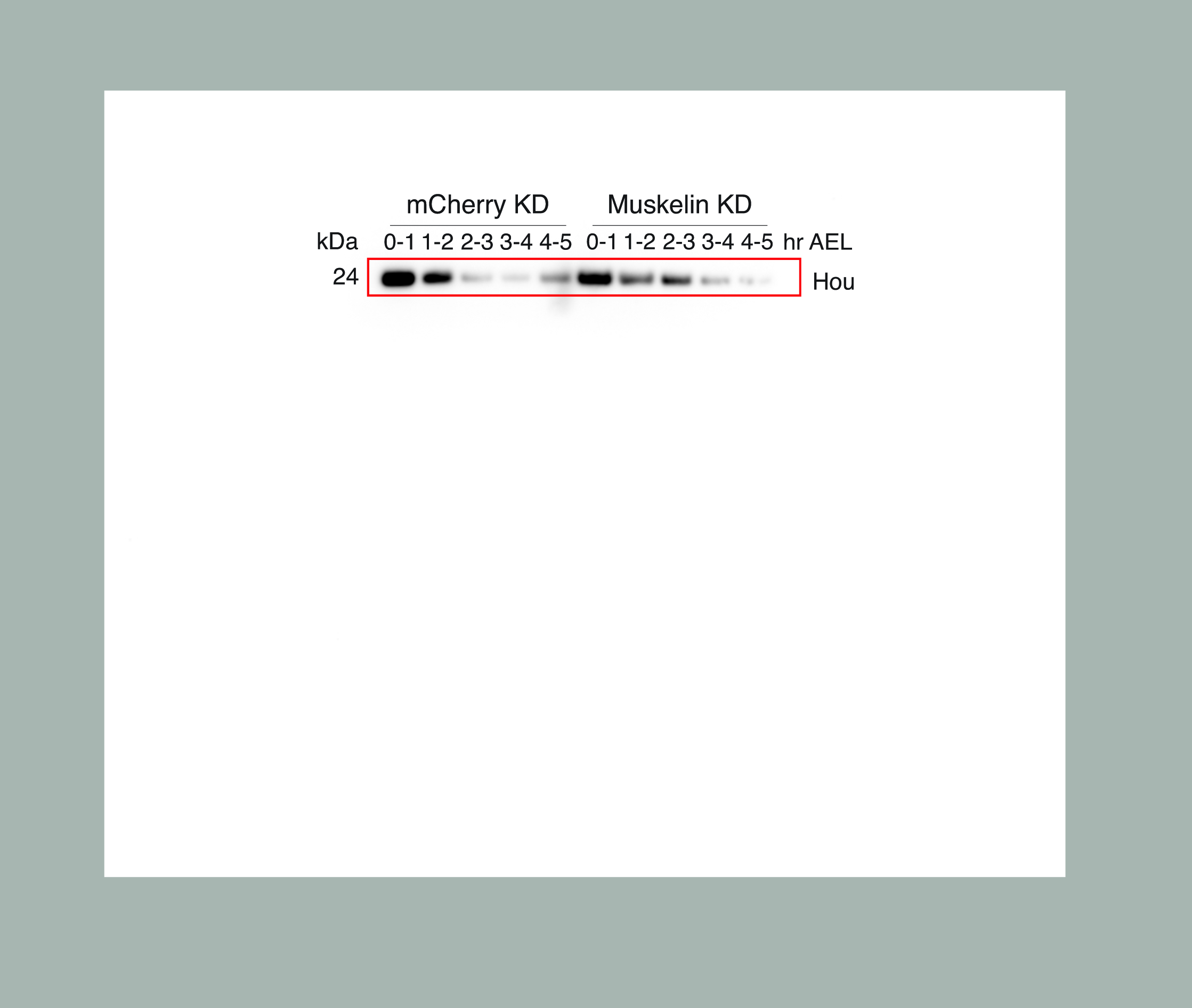

Supplement: Supplementary file 11 — Source data Fig. 3 [file 44319_2025_397_MOESM11_ESM.zip › Figure 3/A/western hou.tif]

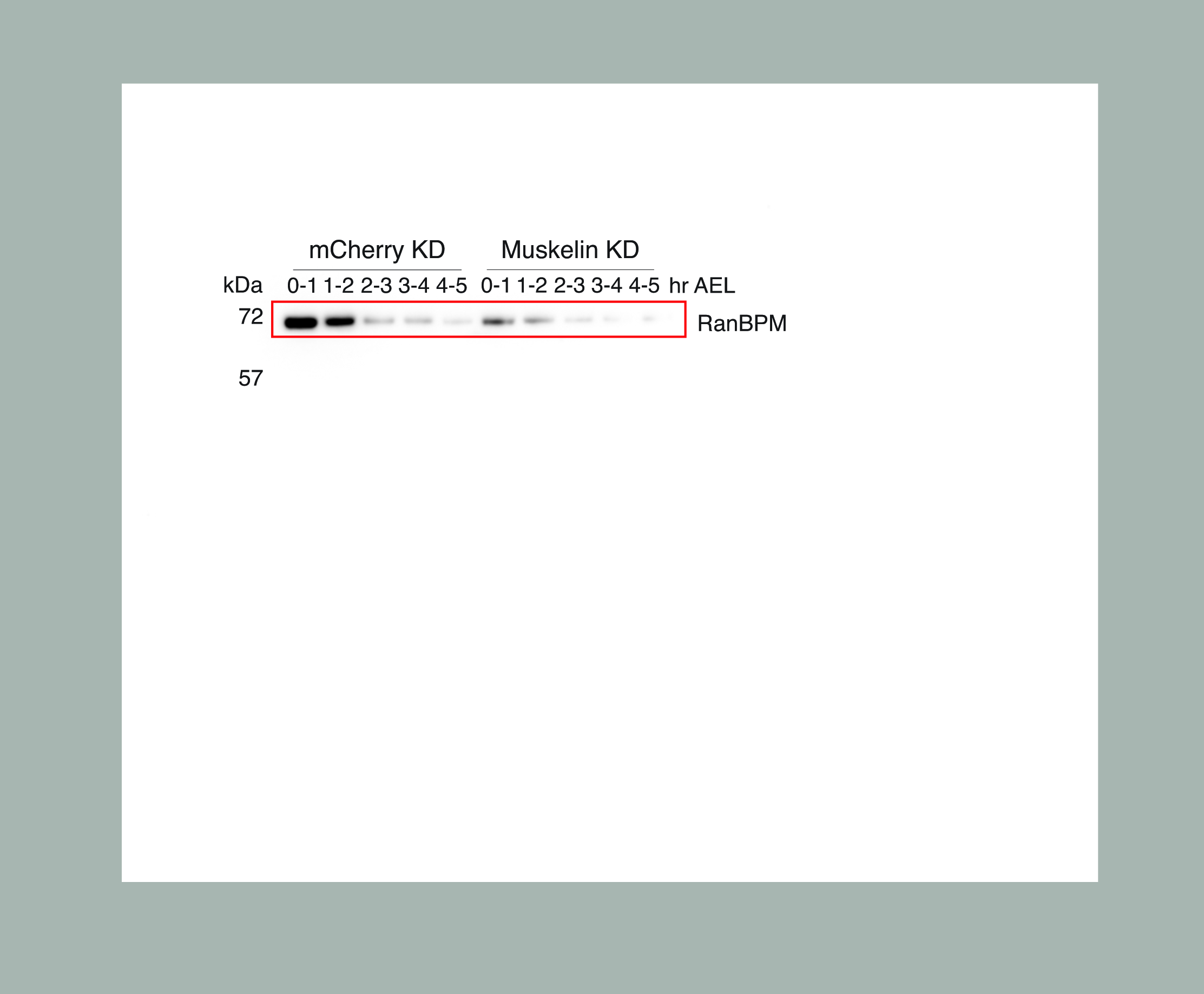

Supplement: Supplementary file 11 — Source data Fig. 3 [file 44319_2025_397_MOESM11_ESM.zip › Figure 3/A/western ranbpm.tif]

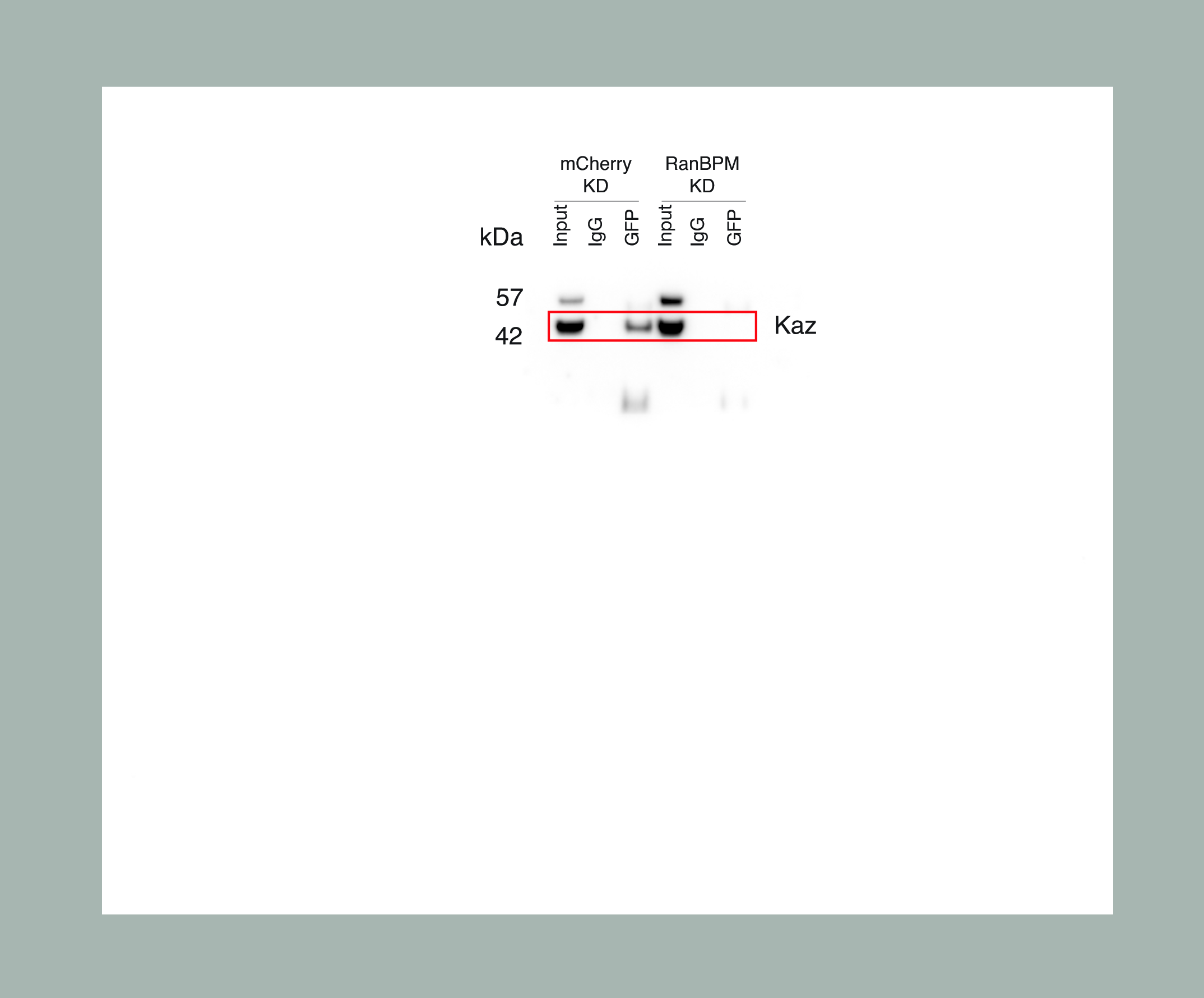

Supplement: Supplementary file 11 — Source data Fig. 3 [file 44319_2025_397_MOESM11_ESM.zip › Figure 3/C/western kaz.tif]

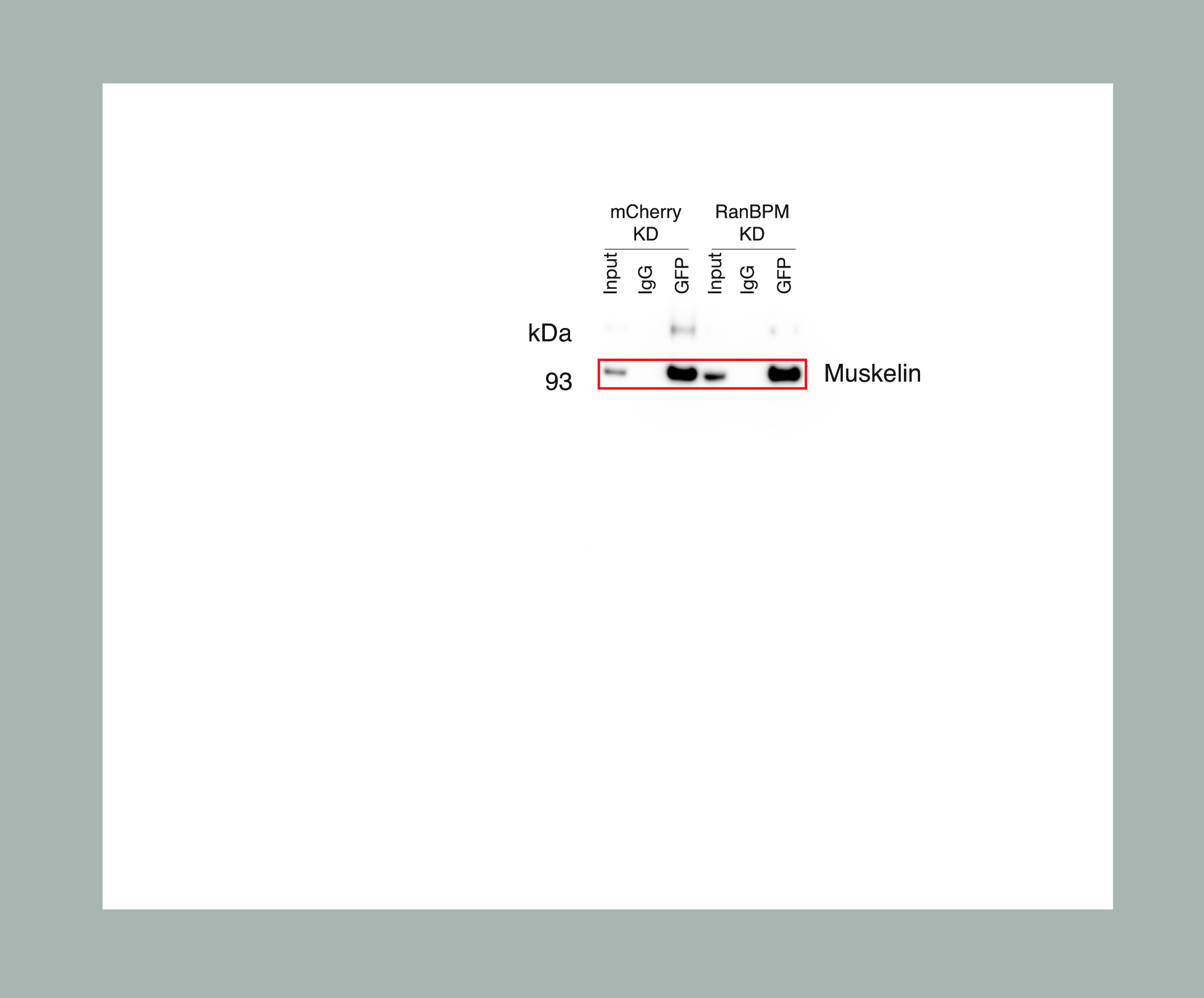

Supplement: Supplementary file 11 — Source data Fig. 3 [file 44319_2025_397_MOESM11_ESM.zip › Figure 3/C/western muskelin.tif]

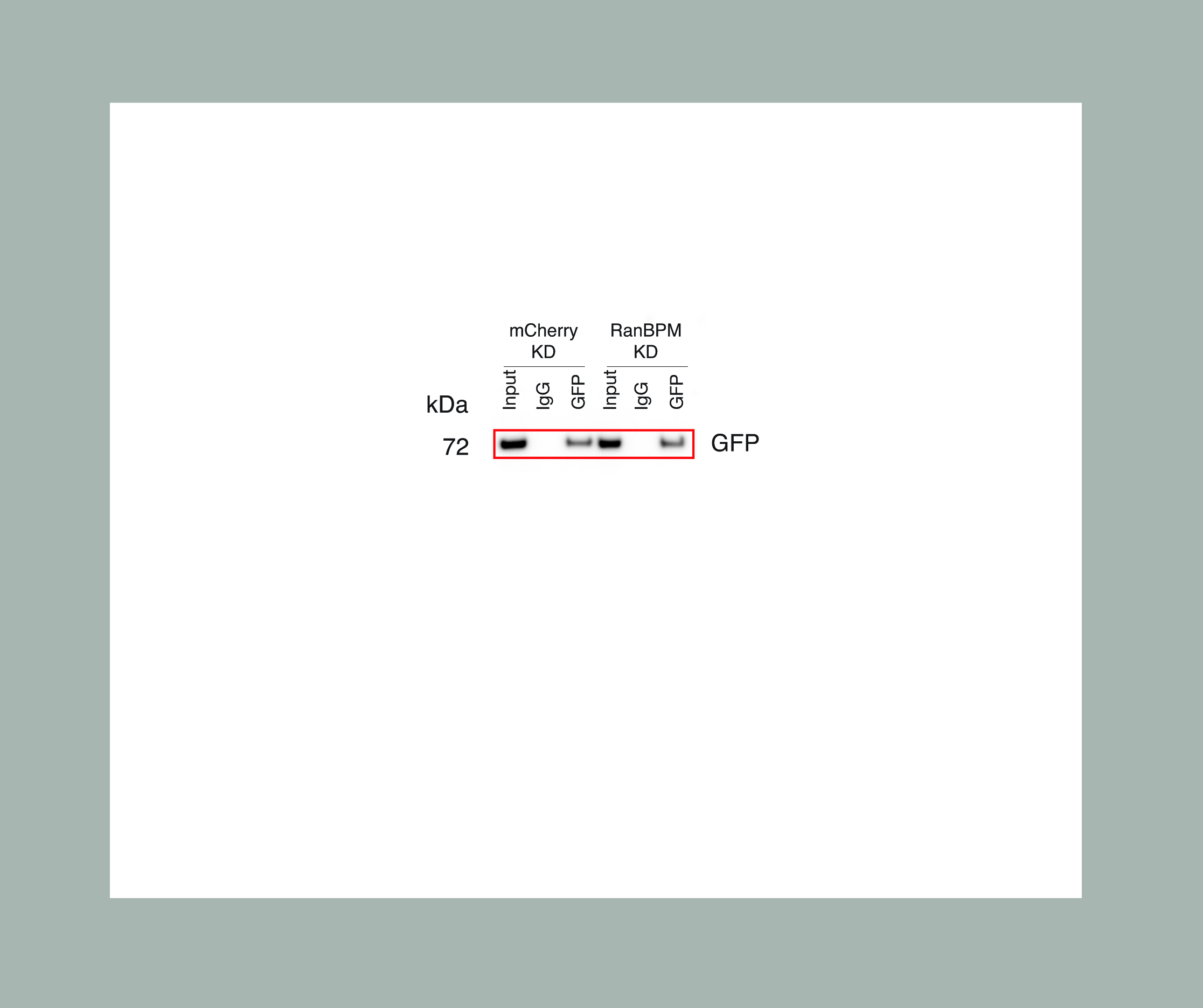

Supplement: Supplementary file 11 — Source data Fig. 3 [file 44319_2025_397_MOESM11_ESM.zip › Figure 3/C/western gfp.tif]

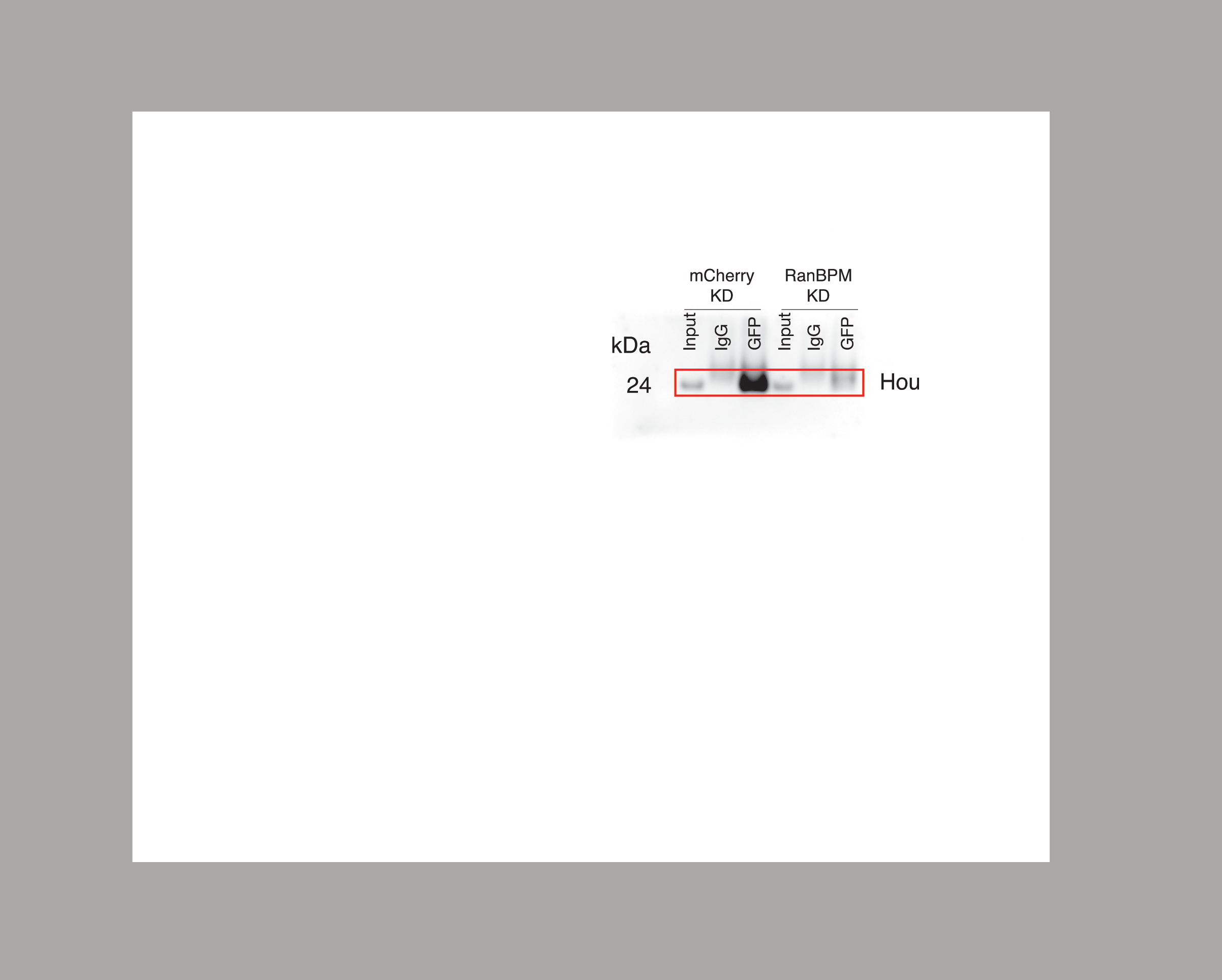

Supplement: Supplementary file 11 — Source data Fig. 3 [file 44319_2025_397_MOESM11_ESM.zip › Figure 3/C/western hou.tif]

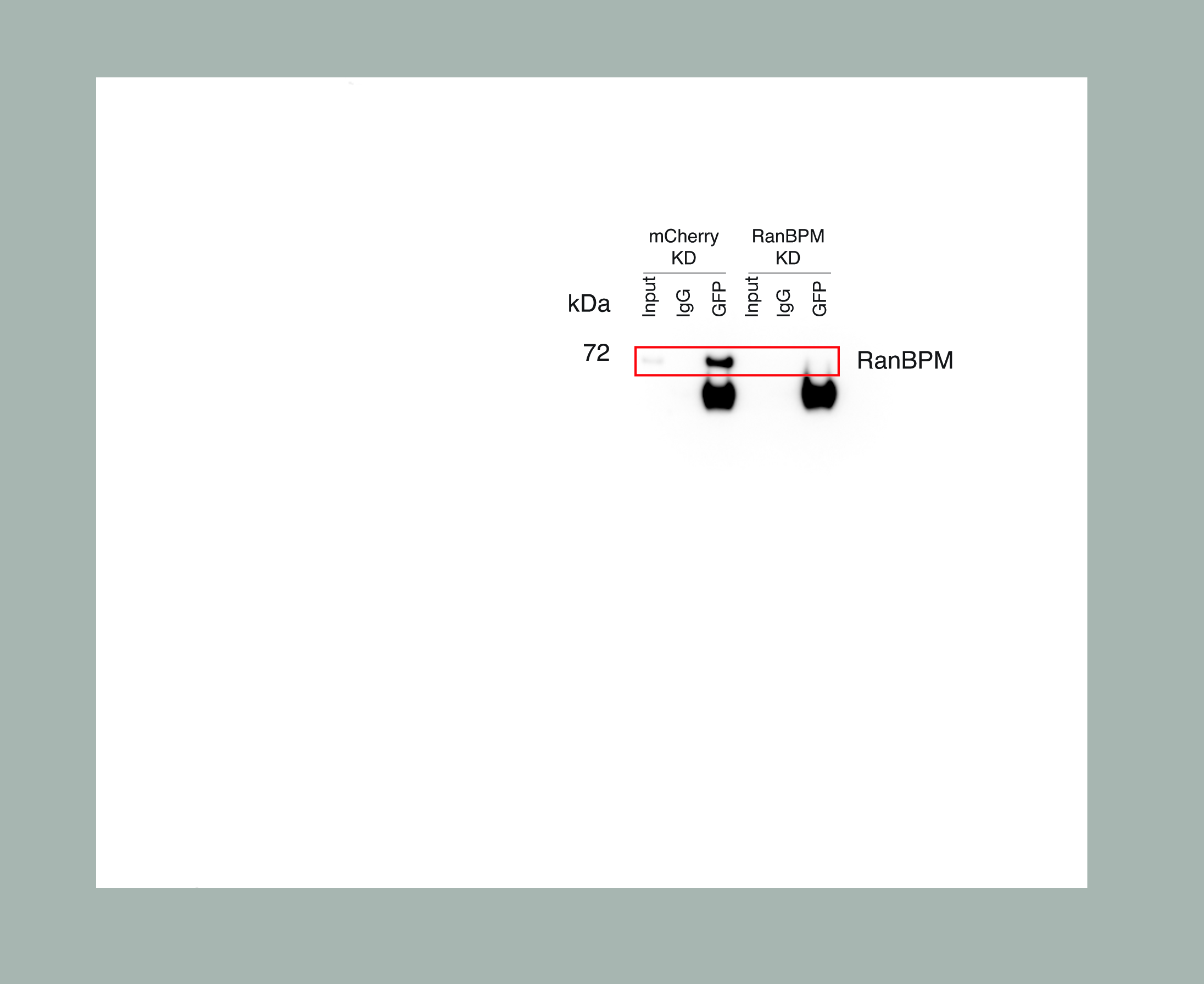

Supplement: Supplementary file 11 — Source data Fig. 3 [file 44319_2025_397_MOESM11_ESM.zip › Figure 3/C/western ranbpm.tif]

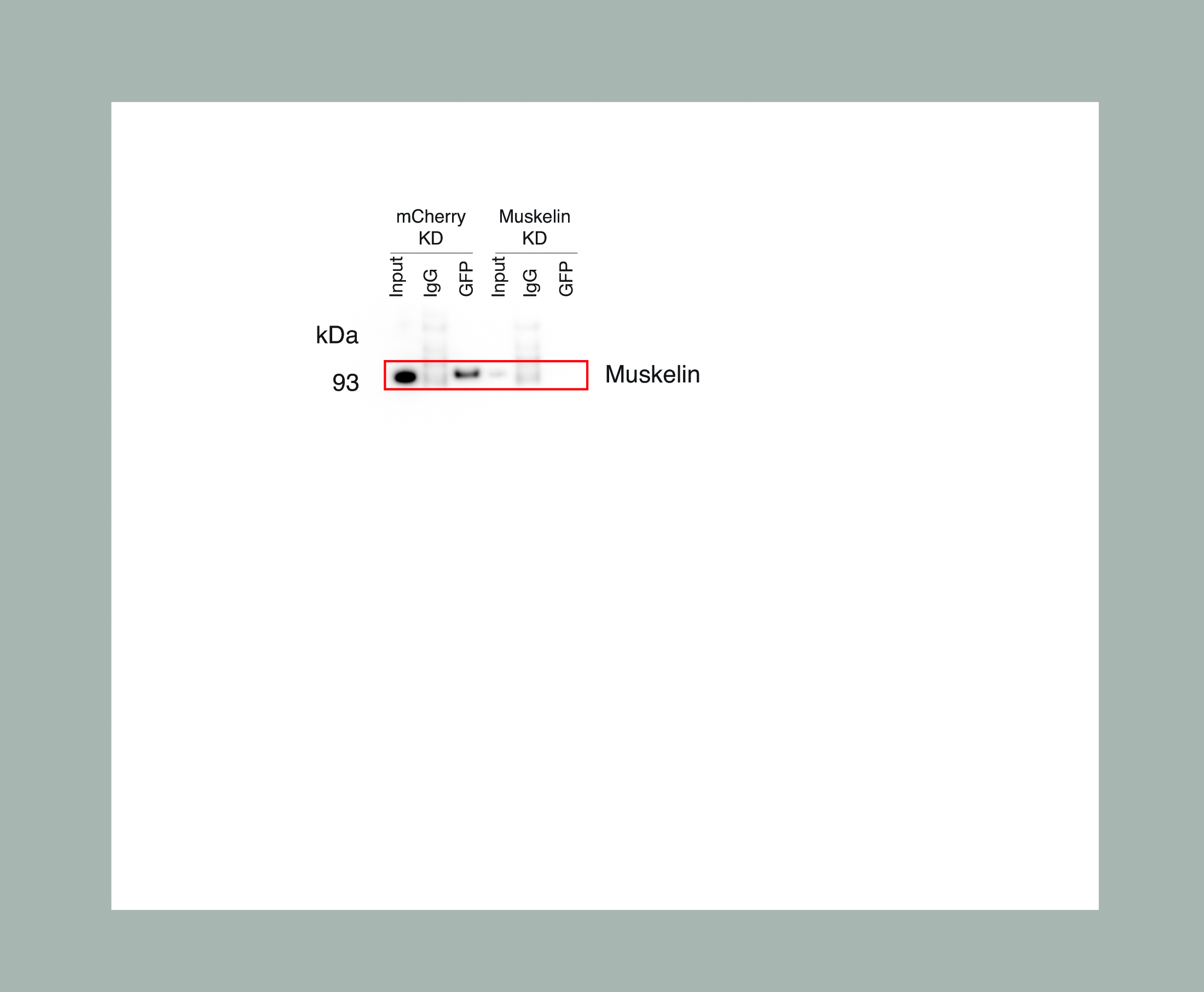

Supplement: Supplementary file 11 — Source data Fig. 3 [file 44319_2025_397_MOESM11_ESM.zip › Figure 3/B/western muskelin.tif]

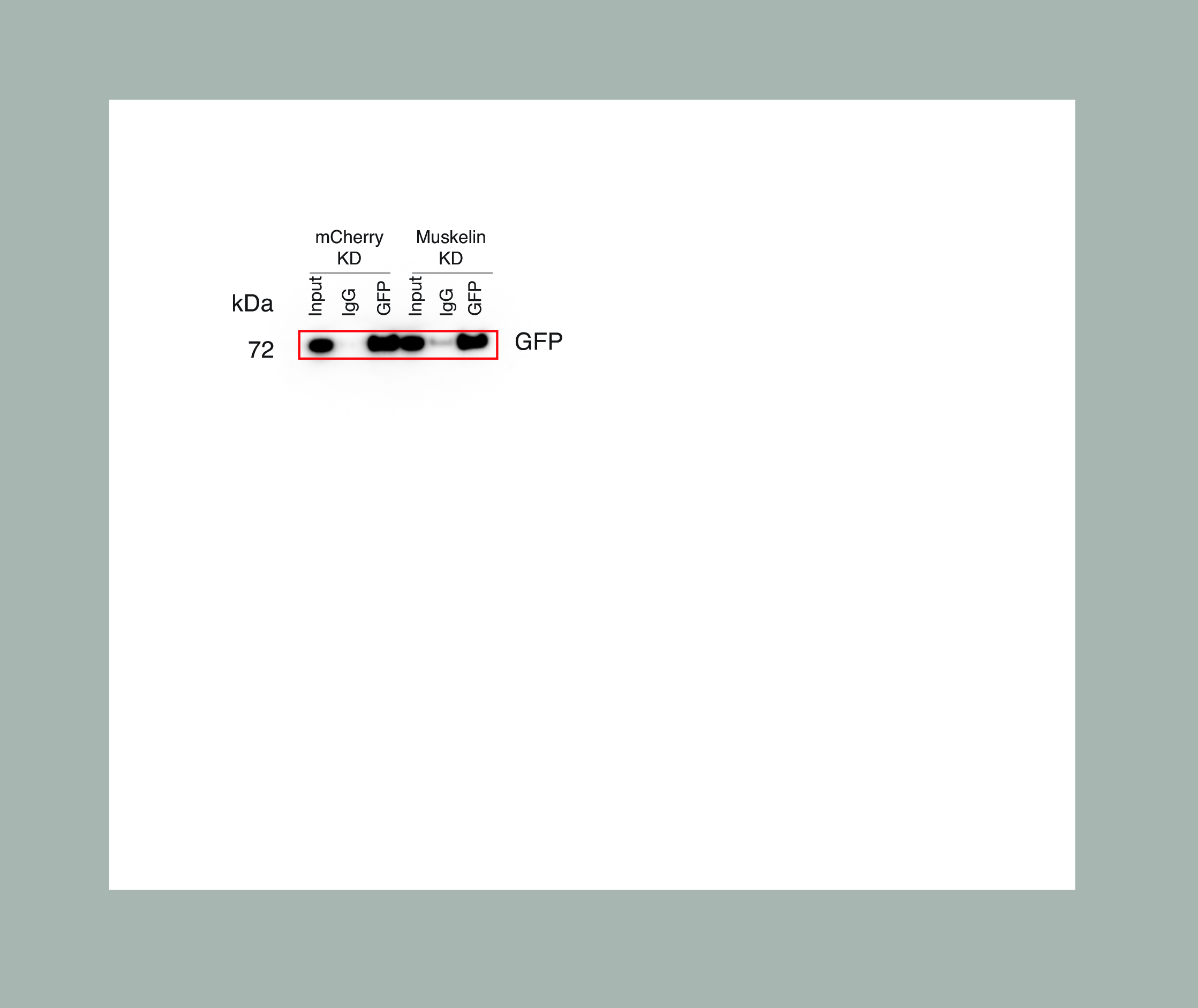

Supplement: Supplementary file 11 — Source data Fig. 3 [file 44319_2025_397_MOESM11_ESM.zip › Figure 3/B/western gfp.tif]

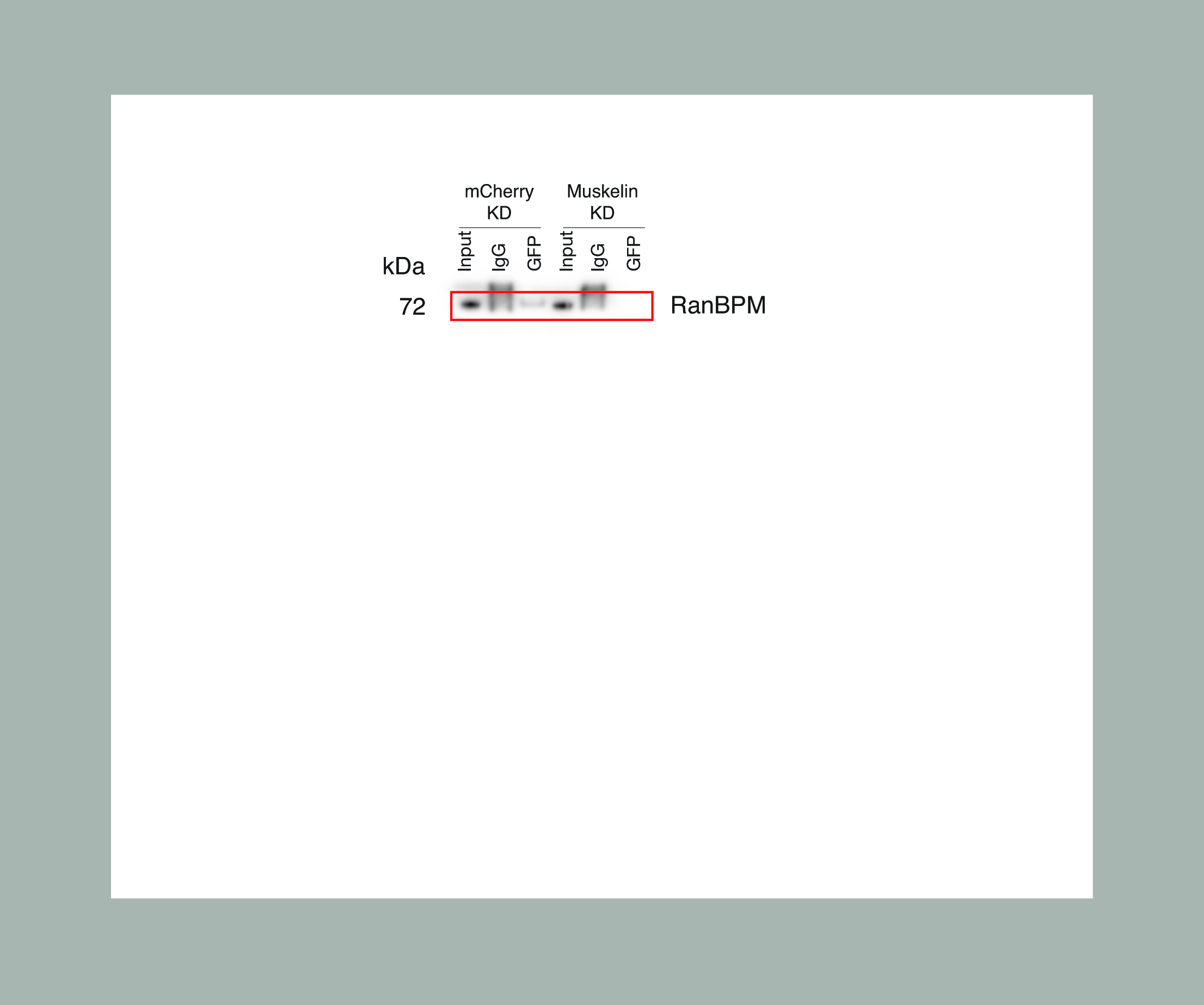

Supplement: Supplementary file 11 — Source data Fig. 3 [file 44319_2025_397_MOESM11_ESM.zip › Figure 3/B/western ranbpm.tif]

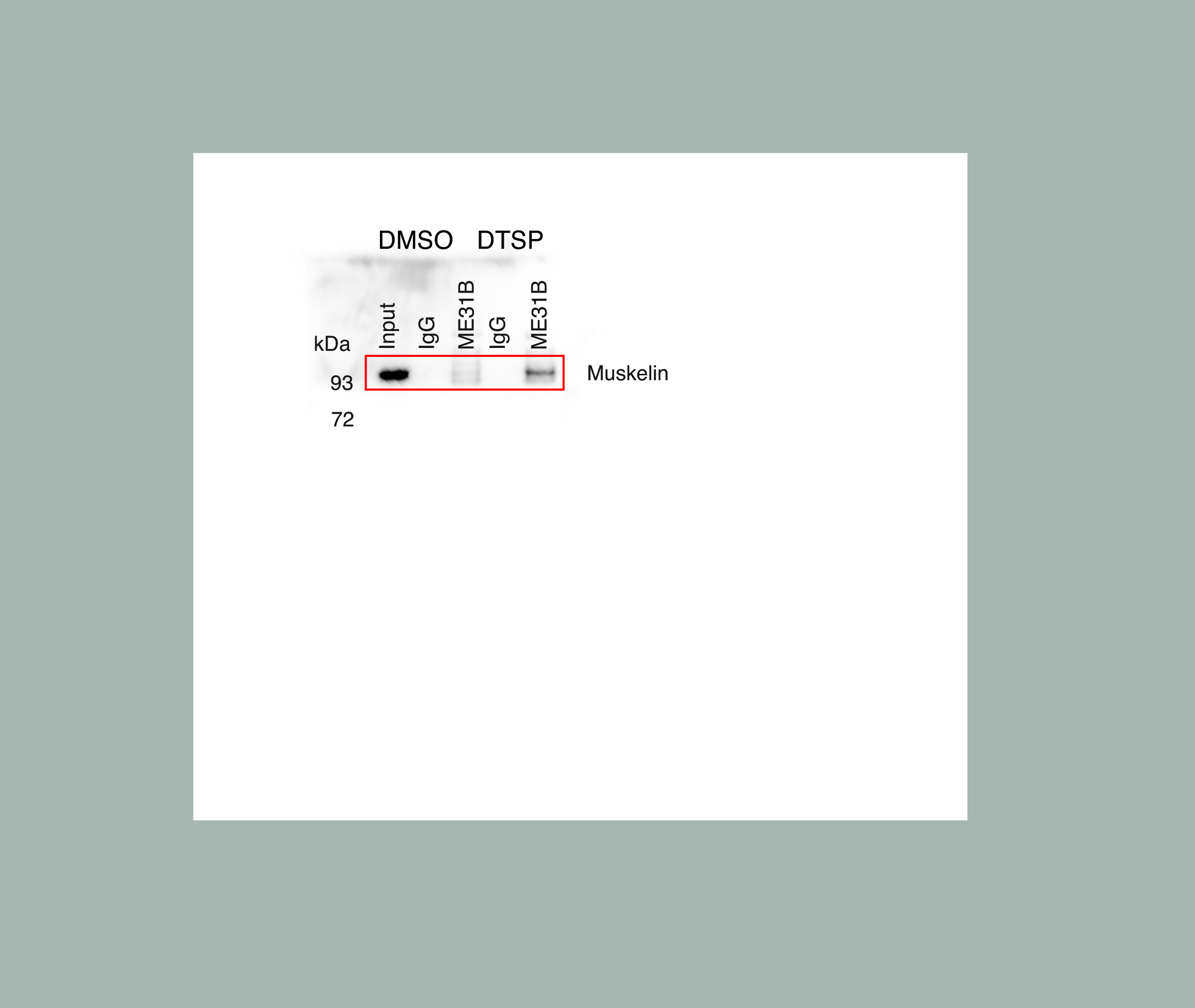

Supplement: Supplementary file 12 — Source data Fig. 4 [file 44319_2025_397_MOESM12_ESM.zip › Figure 4/F/western muskelin.tif]

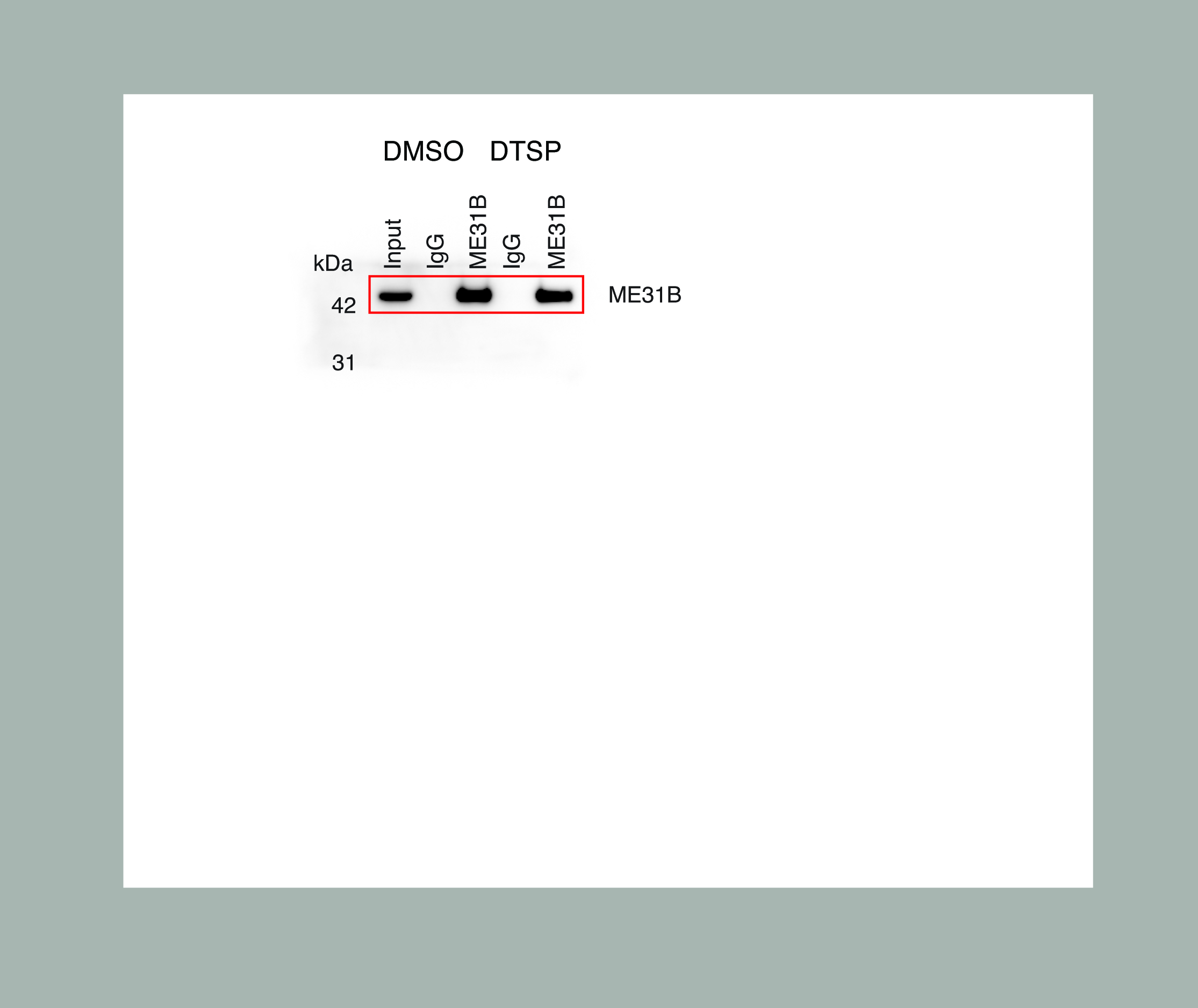

Supplement: Supplementary file 12 — Source data Fig. 4 [file 44319_2025_397_MOESM12_ESM.zip › Figure 4/F/western me31b.tif]

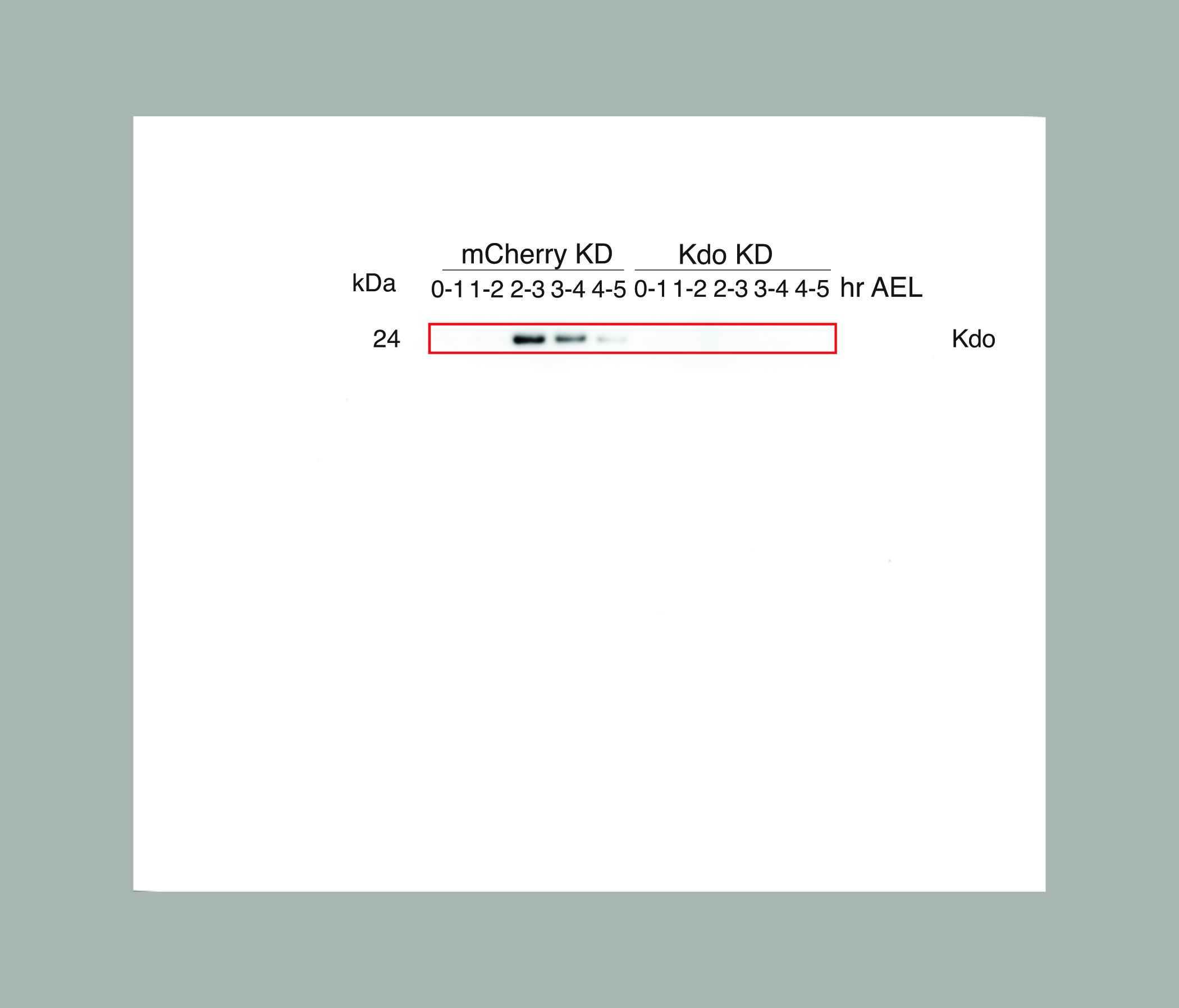

Supplement: Supplementary file 13 — Source data Fig. 5 [file 44319_2025_397_MOESM13_ESM.zip › Figure 5/J/western kdo-.tif]

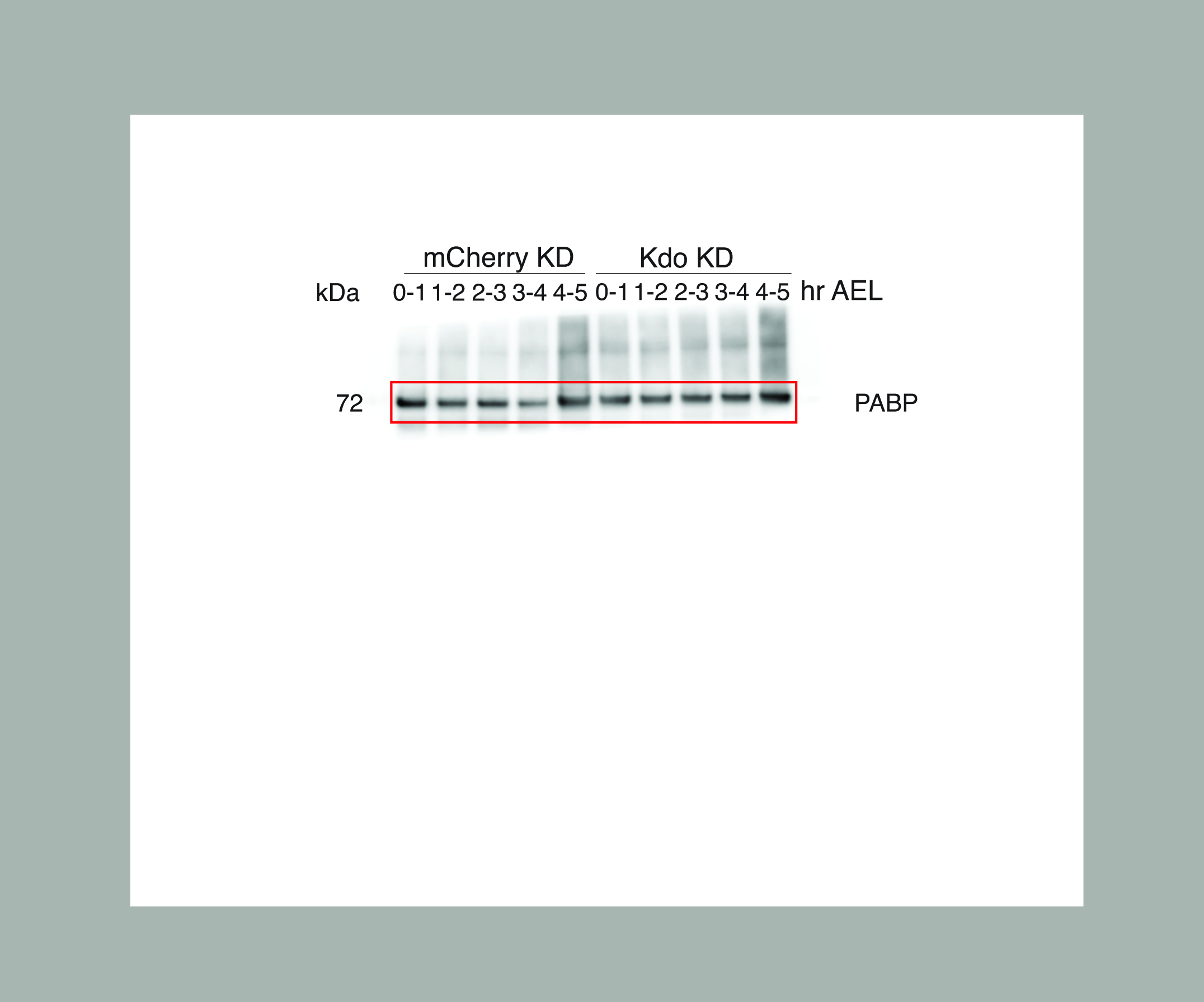

Supplement: Supplementary file 13 — Source data Fig. 5 [file 44319_2025_397_MOESM13_ESM.zip › Figure 5/J/western pabp.tif]

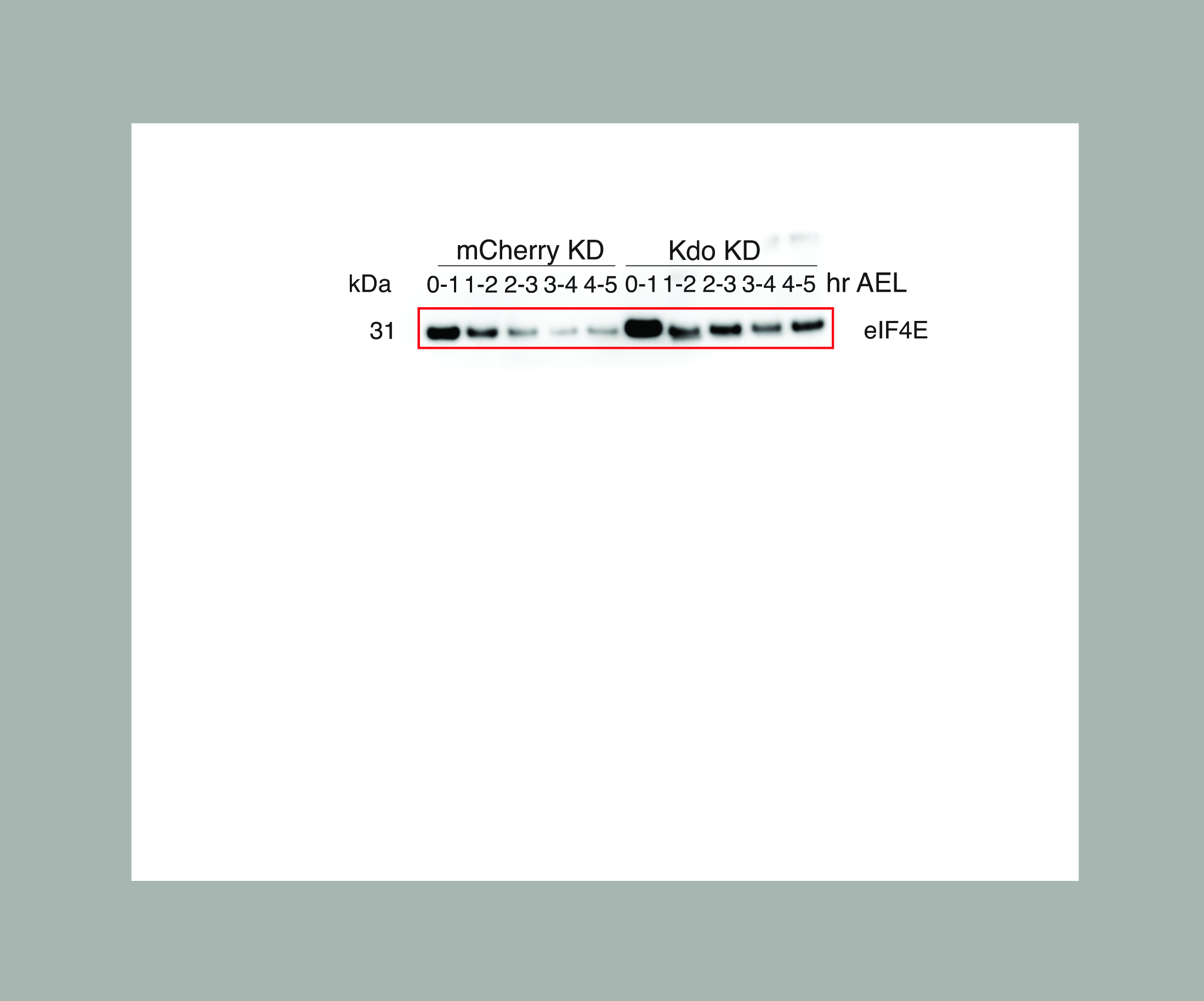

Supplement: Supplementary file 13 — Source data Fig. 5 [file 44319_2025_397_MOESM13_ESM.zip › Figure 5/J/western eif4e.tif]
