# Supplementary material for: Muskelin is a substrate adaptor of the highly regulated Drosophila embryonic CTLH E3 ligase
Source: EMBO Rep. 2025 Feb 20;26(6):1647–69. doi: 10.1038/s44319-025-00397-6 (PMC11933467; doi:10.1038/s44319-025-00397-6)
Supplement: Supplementary file 14 — Expanded View Figures [file 44319_2025_397_MOESM14_ESM.pdf]

Expanded View Figures

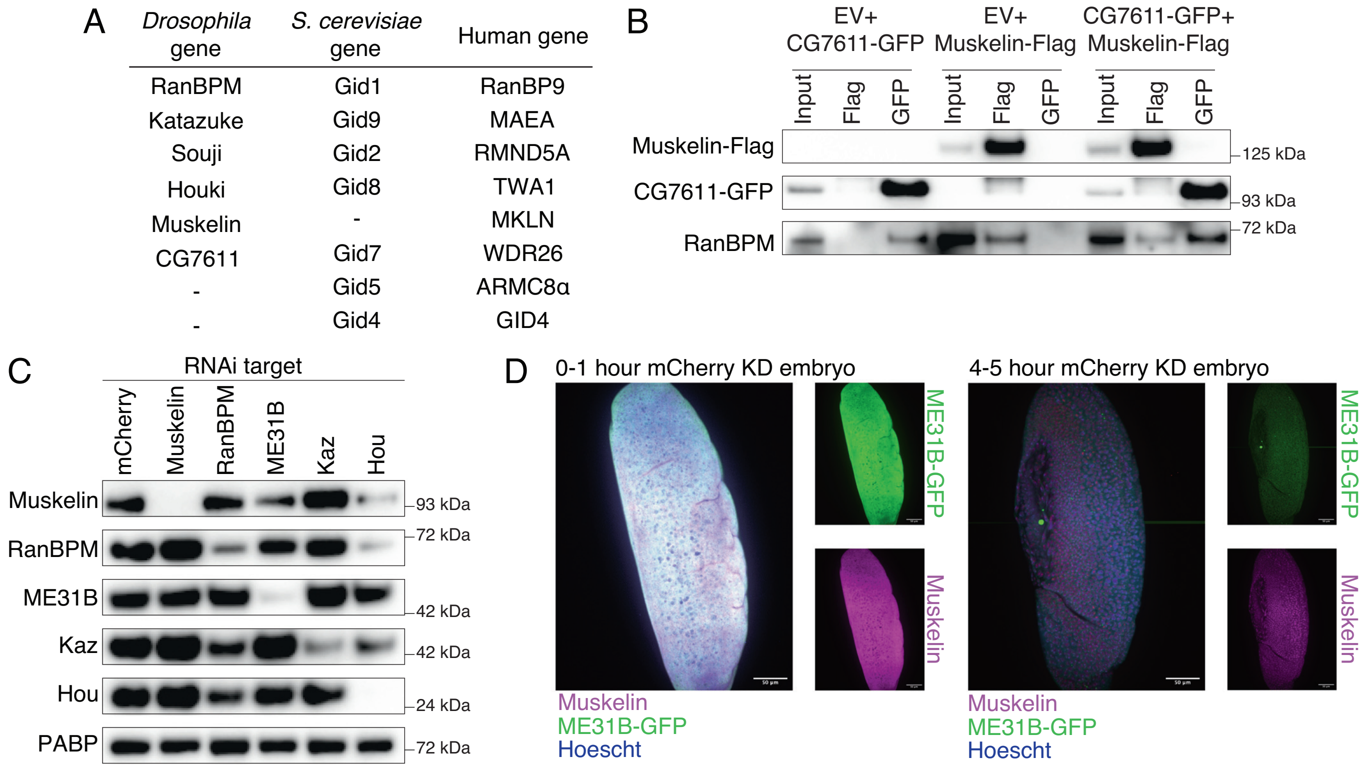

Figure EV1. Gene name comparisons, tool validation, and co-expression analyses.

(**A**) Gene name comparisons from yeast, human, and fly CTLH complex homologs. (**B**) CG7611 and Muskelin are mutually exclusive in their binding to RanBPM in S2 cells. CG7611-GFP and/or Muskelin-Flag were transfected into S2 cells and lysates were immunoprecipitated with anti-Flag beads or anti-GFP. (**C**) Antibody validation. Protein targets were depleted by RNAi in S2 cells followed by western blotting for the target using newly generated antibodies. Gene names above the blots indicate RNAi target, gene names next to blot indicate western blot target. PABP is used as a loading control. (**D**) Immunofluorescence for ME31B-GFP and Muskelin in embryos shows similar localization patterns to immunoprecipitations. 0–1 h and 4–5 h mCherry knockdown (control) embryos were stained with anti-GFP and anti-Muskelin. The signal is diffuse, overlapping, and strong in 0–1 h embryos and decreases noticeably in 4–5 h embryos. Scale bar = 50 μm. Western blots and microscopy images are representative images of three biological replicates.

© The Author(s)

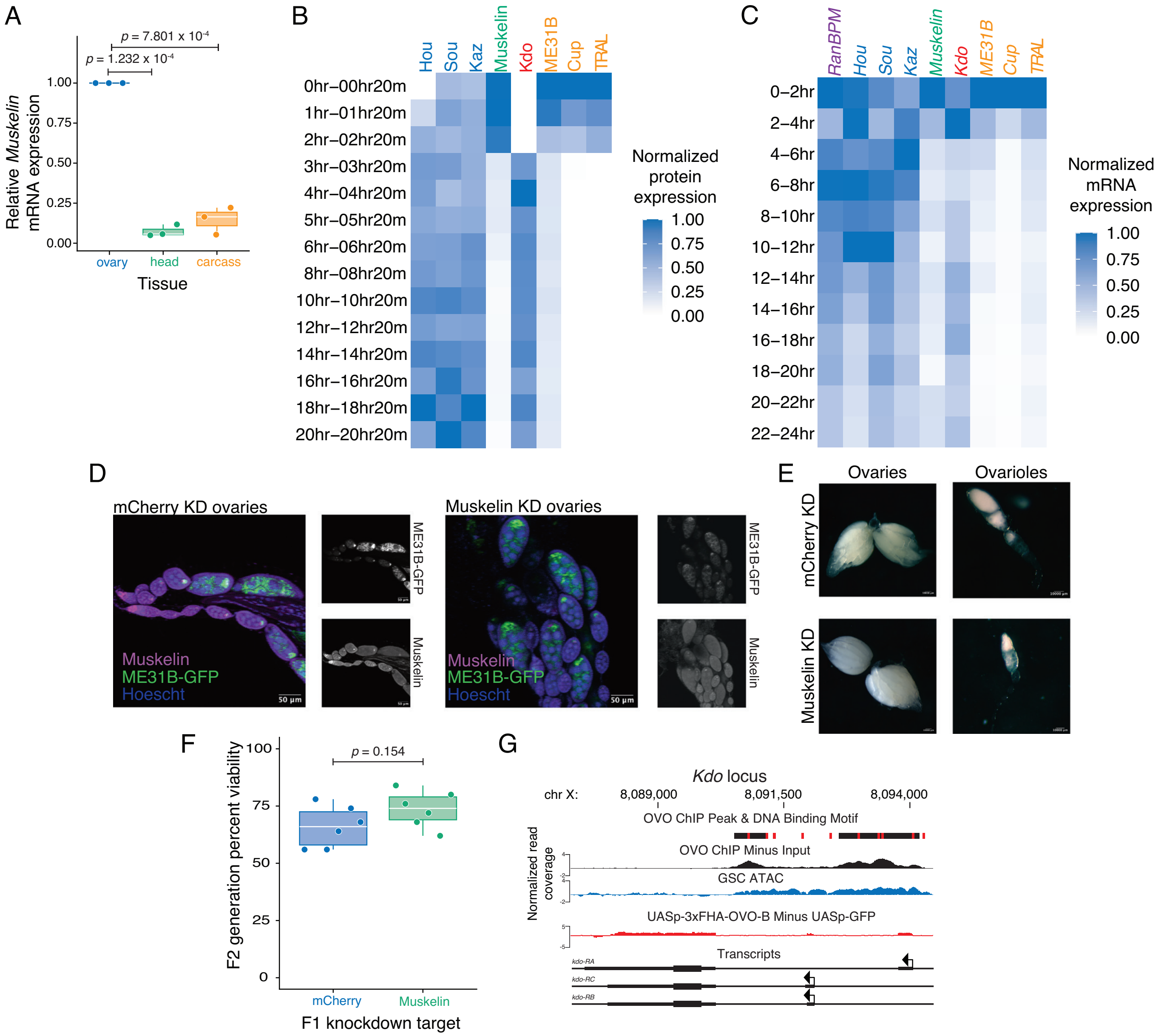

Figure EV2. ***Muskelin*** **expression confirmation, developmental CTLH complex component and target gene expression, immunofluorescence confirmation, Muskelin knockdown phenotypes, and *Kdo* transcriptional control.**

(**A**) qPCR confirmation of *Muskelin* mRNA expression in ovary, head, and carcass, normalized to *Muskelin* expression in ovary. mRNA was extracted from each tissue, converted to cDNA, and amplified using gene-specific primers. Expression levels were compared with a Student's *t* test, $n = 3$, $P = 1.232\times10^{-4}$ comparing ovary to head expression; $P = 7.081\times10^{-4}$ comparing ovary to carcass expression. Boxplot for *Muskelin* mRNA expression in the head: minimum = 0.00183, maximum = 0.0364, median = 0.00565, upper bound = 0.0210, lower bound = 0.00374, mean = 0.0146. Boxplot for *Muskelin* mRNA expression in the carcass: minimum = 0.00378, maximum = 0.0898, median = 0.0605, upper bound = 0.0752, lower bound = 0.0321, mean = 0.0514. (**B, C**) Embryo protein (**B**) and mRNA (**C**) expression of CTLH components and targets is highest early in development. Each gene was normalized to its maximum expression at each timepoint. (**D**) Muskelin immunofluorescence signal is specific to Muskelin expression. Immunofluorescence was performed on Muskelin-depleted ovaries that express ME31B-GFP. Images are representative of $n = 3$ biological replicates. Scale bar = 50 µm. This mCherry knockdown image is the same image as is used in Fig. 2D. (**E**) Brightfield images of mCherry knockdown or Muskelin knockdown ovaries. There are mild phenotypic differences between control and Muskelin knockdown ovaries or ovarioles. Images are representative of $n = 4$ biological replicates. Scale bar = 10,000 µm. (**F**) F2 viability studies for Muskelin knockdown embryos. F2 embryos from F1 mCherry or Muskelin knockdown parents do not show differences in overall viability. $N = 6$, $P = 0.154$ by Welch two-sample t test. Boxplot for mCherry knockdown F2 percent viability: minimum = 56, maximum = 78, median = 66, upper bound = 72.5, lower bound = 58, mean = 66. Boxplot for Muskelin knockdown F2 percent viability: minimum = 62, maximum = 84, median = 74, upper bound = 79, lower bound = 69, mean = 73.67. (**G**) *Kdo* gene level read coverage tracks for OVO ChIP minus input, GSC ATAC-seq, and $ovo^{\Delta BP}/ovo^{ovo\text{-}GAL4}$; *UASp-3xFHA-OVO-B* minus $ovo^{\Delta BP}/ovo^{ovo\text{-}GAL4}$; *UASp-GFP* RNAseq. Red and black rectangles represent significant OVO DNA binding motifs and OVO ChIP peaks, respectively. Gene models are represented at bottom. Small rectangles represent untranslated regions, large rectangles represent translated regions. Arrows indicate transcriptional start sites.

© The Author(s)

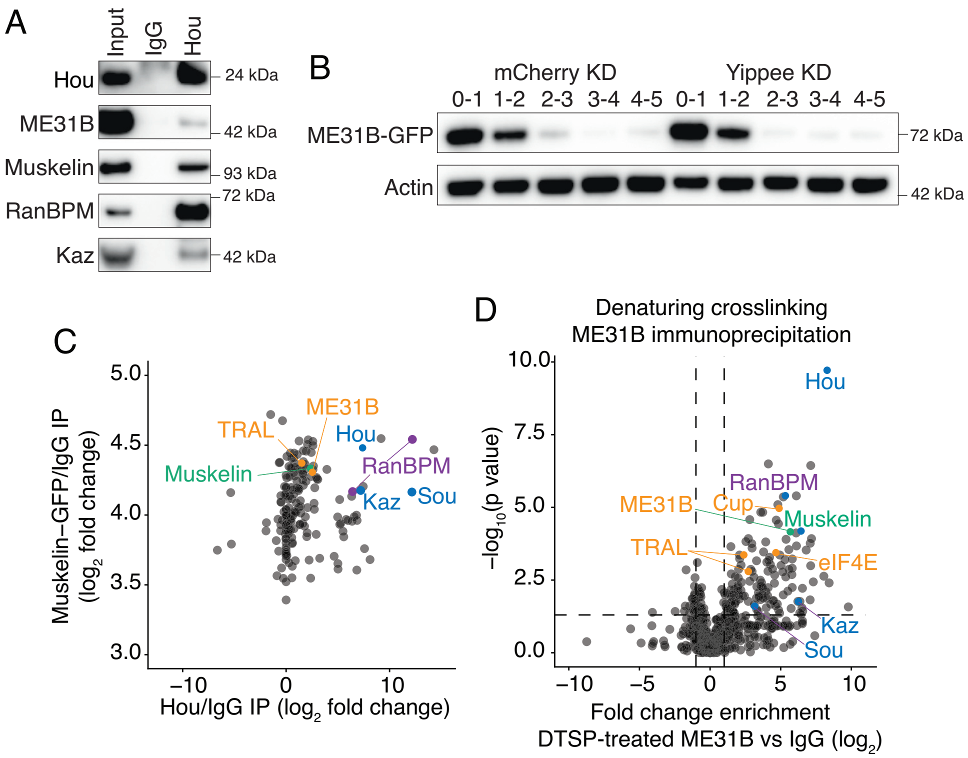

Figure EV3. Hou immunoprecipitation, Yippee knockdown, and denaturing crosslinking ME31B immunoprecipitation mass spectrometry.

(**A**) Hou immunoprecipitation with the newly developed antibody enriches for CTLH components. Hou was immunoprecipitated from *w1118* 1–2 h embryo lysate and probed for CTLH complex components. (**B**) Yippee knockdown does not stabilize ME31B-GFP. Embryo lysates were collected across the MZT time course from control embryos or embryos lacking Yippee and probed for ME31B-GFP and actin (as a loading control). (**C**) No putative substrate adaptors are identifiable from comparing Hou immunoprecipitations to Muskelin-GFP immunoprecipitations. Hou immunoprecipitation fold changes compared to control immunoprecipitation were compared to Muskelin-GFP over control fold changes from Cao et al, 2020. In all plots, blue points represent CTLH complex components, orange points represent targets, green points represent bait protein. Each point represents the average fold change of a specific gene across $n = 3$ biological replicates. (**D**) Denaturing cross-linking ME31B immunoprecipitation mass spectrometry comparing cross-linked ME31B immunoprecipitation to cross-linked control immunoprecipitation demonstrates CTLH component and repressor complex components as close-proximity binding partners of ME31B in 0–1 h *w1118* embryo lysate. Fold changes of ME31B IP in lysates treated with DTSP were compared to IgG IP in lysates treated with DTSP and plotted against the *P* value by *t* test. All western blots are representative of three biological replicates.

© The Author(s)

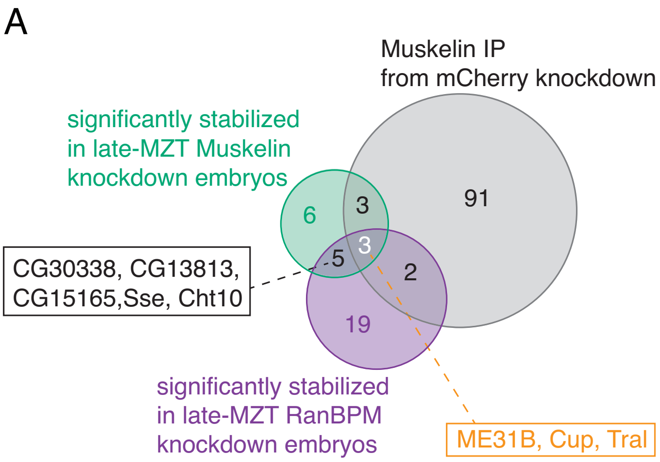

Figure EV4. Venn diagram comparing significantly stabilized proteins from knockdown experiments with Muskelin interactors from immunoprecipitation.

(**A**) Venn diagram comparing significantly stabilized proteins (fold change >2, *P* value, 0.05) from Muskelin knockdown embryos, RanBPM knockdown embryos, and Muskelin immunoprecipitation from mCherry knockdown embryos. Only ME31B, Cup, and Tral are common to all three datasets, highlighting Muskelin's target specificity. Data from this study (Datasets EV3 and EV6).
